# Prehistoric human migration between Sundaland and South Asia was driven by sea-level rise

**DOI:** 10.1038/s42003-023-04510-0

**Published:** 2023-02-04

**Authors:** Hie Lim Kim, Tanghua Li, Namrata Kalsi, Hung Tran The Nguyen, Timothy A. Shaw, Khai C. Ang, Keith C. Cheng, Aakrosh Ratan, W. Richard Peltier, Dhrubajyoti Samanta, Mahesh Pratapneni, Stephan C. Schuster, Benjamin P. Horton

**Affiliations:** 1 Asian School of the Environment, Nanyang Technological University, 50 Nanyang Avenue, N2-01c-63, 639798 Singapore, Singapore; 2 Singapore Centre for Environmental Life Sciences Engineering, Nanyang Technological University, 60 Nanyang Drive, SBS-01n-27, 637551 Singapore, Singapore; 3 GenomeAsia 100K Consortium, 8 Eu Tong Sen Street #14-94, 059818 Singapore, Singapore; 4 Earth Observatory of Singapore, Nanyang Technological University, 50 Nanyang Avenue, N2-01a-15, 639798 Singapore, Singapore; 5 The Jake Gittlen Laboratories for Cancer Research, Penn State College of Medicine, 500 University Drive, Hershey, PA 17033 USA; 6 Division of Experimental Pathology, Department of Pathology, Penn State College of Medicine, 500 University Drive, Hershey, PA 17033 USA; 7 Center for Public Health Genomics, University of Virginia, 1335 Lee Street, West Complex 3rd Floor, MSB 3235, Charlottesville, VA 22903 USA; 8 Department of Physics, University of Toronto, 60 St George Street, Toronto, Ontario M5S 1A7 Canada; 9 Emerge Ventures Pte. Ltd., 8 Eu Tong Sen Street #14-94, 059818 Singapore, Singapore; 10 School of Biological Science, Nanyang Technological University, 60 Nanyang Drive, 637551 Singapore, Singapore

**Keywords:** Population genetics, Genome evolution

## Abstract

Rapid sea-level rise between the Last Glacial Maximum (LGM) and the mid-Holocene transformed the Southeast Asian coastal landscape, but the impact on human demography remains unclear. Here, we create a paleogeographic map, focusing on sea-level changes during the period spanning the LGM to the present-day and infer the human population history in Southeast and South Asia using 763 high-coverage whole-genome sequencing datasets from 59 ethnic groups. We show that sea-level rise, in particular meltwater pulses 1 A (MWP1A, ~14,500–14,000 years ago) and 1B (MWP1B, ~11,500–11,000 years ago), reduced land area by over 50% since the LGM, resulting in segregation of local human populations. Following periods of rapid sea-level rises, population pressure drove the migration of Malaysian Negritos into South Asia. Integrated paleogeographic and population genomic analysis demonstrates the earliest documented instance of forced human migration driven by sea-level rise.

## Abstract (web summary)

Population genomic analyses and palaeogeographical modelling combine to help infer the ancient migration of humans into South Asia from Sundaland, driven by rapid sea level rises.

## Introduction

The transition from the Last Glacial Maximum (LGM; ~26,000–21,000 years ago) to the mid-Holocene (~6000 years ago) was the last major period of global warming in Earth’s history. During this period, the Global Mean Sea Level (GMSL) rose ~135 m^1–3^. This rise in GMSL was characterised by rapid increases over short (decadal and centennial) timescales, termed meltwater pulses (MWPs)^4^, superimposed on a longer-term secular rise^1,2,5,6^. Both short and long-term GMSL rise changed coastal landscapes, not only in northern and southern hemispheres but also in equatorial regions of Southeast Asia. The Sundaland continental shelf, which was exposed as a large landmass including the present-day Malay Peninsula, Sumatra, Borneo and the Philippines, for over 50,000 years before the transition from the LGM^7–10^, was impacted by flooding and submerging of large areas.

Modern humans have inhabited the exposed Sundaland continental shelf since ~70,000–50,000 years ago^11^. The present-day descendants of these early inhabitants are the indigenous tribes of the Andaman Islands, Malay Peninsula, Thailand, and Philippines, referred to as Andamanese, Malaysian and Philippine Negritos, respectively^12,13^. Locally, these tribes are referred to as Orang Semang in Malaysia, whereas Aeta and Ati groups are part of indigenous groups living in the Philippines^14–19^. Archaeological data indicates that these indigenous tribes have continuously inhabited the Malay Peninsula^20–22^. Previous studies suggest that climate changes since the LGM have influenced populations living in the Sundaland by inferring population history mostly based on mitochondrial DNA^23–25^ or Y chromosome data^26,27^, and genotyping data^28^. However, only the level of resolution provided by whole-genome sequence datasets allows the study of the unbiased demographic history of indigenous populations inhabiting Sundaland before and during the post-LGM sea-level rise.

Here, we combine the reconstruction of 1) sea-level rise since the LGM to produce highly resolved spatial and temporal paleogeographic maps of Southeast and South Asia; and 2) the human demographic history inferred using high-depth, human whole-genome sequence datasets generated from an extensive set of ethnicities of Southeast and South Asia by the GenomeAsia 100 K consortium^29^. The natural history of the Sundaland region demonstrates the impact of rising sea levels on pre-historic, equatorial human populations. Southeast and South Asian regions are particularly suited for understanding the impact due to the long-standing modern human occupation. Importantly, Southeast Asia is the only region globally that experienced major reductions in the land area during the transition from the LGM, and at the same time, has continuously been occupied by today’s indigenous human populations. Hence, our high-resolution paleogeographic/population genomic study outlines the impact of paleoclimate changes on past and present human demography.

## Results

### Rapid sea-level rise in Southeast Asia

**Fig. 1:**
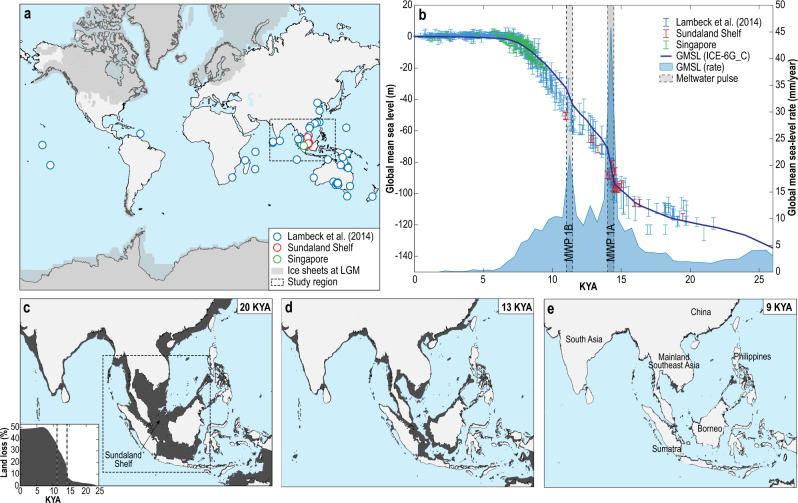
Global mean sea level (GMSL) and paleotopography maps of land cover change since the Last Glacial Maximum (LGM). **a** Distribution of sites used to constrain GMSL in **b** since the LGM^1^ showing study region and ice sheet extent at the LGM from the ICE-6G_C model^2,30^. **b** Sea-level data from sites in **a** including Sundaland Shelf (red) and Singapore (green) and magnitude and rate of GMSL change from ICE-6G_C showing the timing of meltwater pulse (MWP) 1 A and 1B. Paleotopography maps of land cover change showing former coastline (dark grey) at **c** 20 thousand years ago (KYA), **d** 13 KYA and **e** 9 KYA. The black dashed line in **c** indicates the area used to calculate land loss (%) in inset. The base maps were retrieved from the public dataset^83^ with open access.

Using the ICE-6G_C global ice history model^2,30^ and HetM-LHL140 3D Earth model^31,32^ we infer the rate of sea-level rise in Southeast and South Asia at 500-year increments. The resulting paleotopographic maps cover a timespan of 26,000 years ago to the present (Fig. 1, Supplementary Movie). GMSL is shown to have risen from ~−122 m to −1 m between 22,000 years ago to 6000 years ago, which was punctuated by two periods of rapid sea-level rise (MWP1A and 1B, Fig. 1b). Between 22,000 and 16,000 years ago, the rate of GMSL rose ≤5 mm/year, subsequently accelerating to a maximum of ~46 mm/year between 14,500–14,000 years ago (MWP1A). GMSL continued to rise at a rate of ~10 mm/year for 3000 years before another rapid increase occurred with a rate of ~22 mm/year between 11,500–11,000 years ago (MWP1B) at the beginning of the Holocene (Fig. 1b). According to our model, during the early Holocene, the rate of GMSL rise decreased from ~10 to ~3 mm/year. In Southeast and South Asia, the mid-Holocene is characterised by a highstand varying in timing and magnitude^8^. In Singapore, the highstand reached a magnitude of ~4 m 5200 years ago, relative to today’s sea level^33^.

To infer paleogeographic changes in Southeast Asia, we calculated the land cover change since the LGM as a percentage land loss (Fig. 1c–e, Supplementary Table 1). We show that the Sundaland area was reduced ~50% from the LGM to the mid-Holocene (Fig. 1c inset, Supplementary Table 1). In Southeast Asia, the land area reduced ~18% by the end of MWP1A, including the breakage of land bridges between Palawan in Philippines and Borneo, both becoming islands (Fig. 1c inset, Supplementary Movie). The land area was reduced by a further ~19% by the end of MWP1B (Fig. 1c inset) when the land bridges from Sumatra to the Malay Peninsula were broken. Land area reduced to a minimum at the mid-Holocene highstand (6500 years ago). Thereafter, a fall in sea levels towards present-day created a slight increase (~2%) in land area.

### Population structure and admixture

**Fig. 2:**
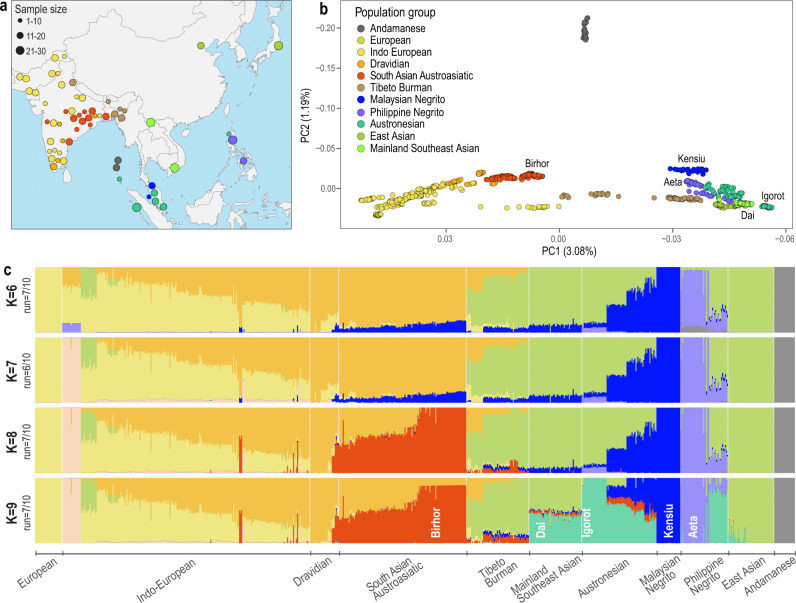
Ancestral population structure in Southeast and South Asia. **a** Geographic locations of the studied populations and their sample sizes included are indicated on the map. The ethnicity label can be found in Supplementary Fig. 1. The map was constructed using ggplot2^100^, and the base map was retrieved through ‘map_data’ package^101,102^. The colour scheme represents the ancestral group as shown in plot. **b** The PCA^34^ result for the 763 genomes are plotted with PC1 and PC2 (percentage of eigenvector). Each dot represents an individual. Each ethnic group is shown as a different colour, and the legend for each ethnic group is in Supplementary Fig. 2. **c** The proportion of ancestries of the 763 individuals estimated using ADMIXTURE^35^ for *K* = 6–9 ancestral components. A bar represents an individual, and a colour indicates an ancestral component. *K* = 7 is the most optimal number of components based on the cross-validation error estimate (Supplementary Fig. 3). The robustness of the clustering is shown next to K. The 11 population groups were classified based on the combination of ancestral components and named under the plot. The South Asian Austroasiatic and Mainland Southeast Asian populations include the Malaysia Negrito ancestry (blue) at *K* = 6–7, which later is included in the new ancestral component (red) at *K* = 8–9. Cross-validation error of the analysis and more results are shown in Supplementary Fig. 3.

High-coverage whole-genome data generated by the GenomeAsia 100 K consortium^29^ for 763 individuals were derived from 59 ethnic groups that are native to Southeast and South Asia with European populations as reference groups (Fig. 2a, Supplementary Fig. 1, and Supplementary Table 2). The genome data were analysed for their population structure and admixture using principal component analysis (PCA)^34^ (Fig. 2b and Supplementary Fig. 2) and ADMIXTURE^35^ (Fig. 2c and Supplementary Fig. 3). Based on the results, the genomes included in our analysis are categorised into 11 population groups (Fig. 2a–c, Supplementary Table 2). For Southeast Asia, we identify five population groups, Andamanese (Jarwa and Onge), Malaysian Negritos (Kensiu and Kintak), Philippine Negritos (Aeta), Austronesians (Igorot, Temuan, Senoi, and indigenous people from Mentawai and Nias) and Mainland Southeast Asians (Dai and Kinh). We further show that the Andamanese and both Negrito groups, except the Ati, display minimum admixture and high homozygosity, suggesting spatial isolation from one another for an extended time (Fig. 2c and Supplementary Figs. 3 and 4). For South Asians, we consider four population groups in South Asia: The Indo-European, Dravidian, Tibeto-Burman and Austroasiatic groups.

Importantly, we identify substantial Malaysian Negrito ancestry in both Mainland Southeast Asians and South Asian Austroasiatic groups (*K* = 6–7 in Fig. 2c). The latter are tribal groups living in East India and are considered to be the earliest inhabitants of India based on genetic studies^36–38^. This genetic contribution of Malaysian Negritos variants to a major South Asian population group is supported by five findings.

First, on the differentiation between South Asians and Southeast Asians reflecting the PC1 in the PCA, South Asian Austroasiatic groups are located closer to the Malaysian Negritos than to other South Asian groups of Indo-Europeans and Dravidians (Fig. 2b).

Second, the Admixture analysis identifies Malaysian Negrito ancestry (blue label in Fig. 2c, *K* = 6–7) in Mainland Southeast Asians (light green label in Fig. 2c), in some Tibeto-Burmans (brown label in Fig. 2c), and in South Asian Austroasiatic groups (red label in Fig. 2c). A comparison of the admixture analysis on the X chromosomes with that on the autosomes shows that the genetic contributions of male and female Malaysian Negritos to the South Asian Austroasiatic groups are different (Supplementary Fig. 3). The proportion of Malaysian Negrito ancestry in the X chromosomes (1.3% ± 2.8) is less than that in the autosomes (7.4% ± 2.9), suggesting a male bias in the potential migration of Malaysian Negritos to South Asia (Supplementary Fig. 3). This gender bias in genetic contributions between the Malaysian Negritos and South Asian Austroasiatic groups is consistent with uniparental genetic lineages (Supplementary Fig. 5). Previous studies have found dispersal signals of the Southeast Asian Y haplogroup in South Asian Austroasiatic groups^26,27,39^.

**Fig. 3:**
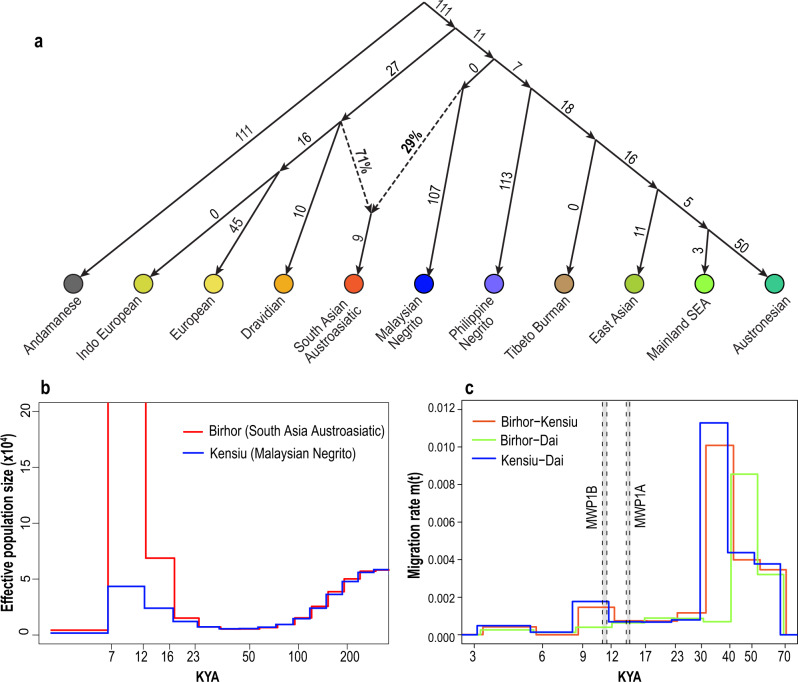
Ancient admixture between South Asian Austroasiatic and Malaysian Negrito groups. **a** The genetic relationships and admixture history for Southeast and South Asian are modelled using qpGraph^41^. The phylogenetic relationship was estimated using Treemix^40^ (Supplementary Fig. 9). The South Asian Austroasiatic group is the admixed population in this model and the two best ancestral populations among the 11 population groups are Dravidians (71%) and Malaysian Negritos (29%) (Supplementary Fig. 10 and Supplementary Table 4). Mainland SEA in the figure is mainland Southeast Asians. **b** The changes in effective population size (*X* axis) over time (*Y* axis) for Birhor and Kensiu are estimated using MSMC. The Birhor shows an extreme increase in the effective population size to over 500,000, 12–7 thousand years ago (KYA on the *X* axis), and the population size is out of the *Y* axis range in the plot (shown the entire plot in Supplementary Fig. 6b). **c** The migration rate between a pair of populations over time is estimated by MSMC-IM^44^. The *X* and *Y* axes indicate the time (KYA) and the migration rate at the time *t*, *m(t)*, respectively. The lines show the estimated migration rate between the three pairs of populations over time as shown in the legend. The periods of the two meltwater pulses (MWP) 1A and 1B are indicated as grey bars.

Third, we reconstructed the admixture history using Treemix^40^ (Supplementary Fig. 9) and qpGraph^41^ which can estimate the best-fitting model for the ancestral populations and their proportions for an admixed population (Fig. 3a). The qpGraph results show that admixture between Dravidian (71%) and Malaysian Negrito (29%) groups is the best combination to represent the South Asian Austroasiatic groups. For the Mainland Southeast Asians, the best fit is the pair of Austronesians (96%) and Malaysian Negritos (4%) (Supplementary Fig. 10 and Supplementary Tables 4 and 5). The large proportion of the Malaysian Negritos in South Asian Austroasiatic groups as compared to Mainland Southeast Asians support again the admixture between the two populations.

Fourth, we used Multiple Sequentially Markovian coalescent (MSMC) analysis^42,43^ to estimate effective population size changes over time. The dramatic increase in population size of South Asian Austroasiatic groups compared to the light gradual increase in population size of Malaysian Negritos suggests the introduction of a distinct lineage into South Asian Austroasiatic groups (Fig. 3b and Supplementary Figs. 5 and 6). Therefore, the direction of admixture between the two populations is likely from Malaysian Negritos to South Asian Austroasiatic groups.

Lastly, we estimated the timing of admixture events based on the migration rate inferred by MSMC-IM^44^. Increasing migration rates observed after the population split of the Kensiu (Malaysian Negritos) and the Birhor (South Asian Austroasiatic) groups indicate admixture between them ~12,000–9000 years ago (Fig. 3c and Supplementary Fig. 7). Similarly, an increase in migration rate between the Kensiu and Dai (Mainland Southeast Asians) groups indicates admixture ~12,000–8000 years ago (Fig. 3c and Supplementary Fig. 7). In contrast, the increase in migration rate was not observed between Birhor and any other Southeast Asian populations—the Dai, Igorot (Austronesians), or Aeta (Philippine Negritos). Therefore, the origin of the admixture found in South Asian Austroasiatic groups is the Malaysian Negritos, rather than Mainland Southeast Asians that contained the Malaysian Negrito ancestry. The admixture between the South Asian Austroasiatic and Malaysian Negrito groups could only have occurred after the split of the Austronesians and Mainland Southeast Asians, ~11,000–10,000 years ago (Supplementary Fig. 6a), since there is no admixture signal between the Kensiu and Igorot. Thus, the timing of the admixture is predicted to be ~10,000–8000 years ago. This estimated time range matches the admixture between Malaysian Negritos and Mainland Southeast Asians. The MSMC-IM results with the Dai are replicable using the Kinh (KHV) population (Supplementary Fig. 7 and Supplementary Table 3).

The MSMC analysis indicates the shaping of population structure in Southeast Asia. Multiple population splits occurred simultaneously across Kensiu (Malaysian Negritos), Aeta (Philippine Negritos), and the common ancestors of Igorot (Austronesians) and Dai (Mainland Southeast Asians), ~15,000–13,000 years ago (coalescence rate = 0.5, Supplementary Fig. 8, and Supplementary Table 3). These splits coincide with rapid sea-level rise MWP1A and the flooding of Sundaland, resulting in the formation of the Philippine archipelago (Fig. 1d, e). Subsequently, the Igorot and Dai split ~11,000–10,000 years ago (Supplementary Fig. 8 and Supplementary Table 3) co-occurred with MWP1B and the submergence of coastal areas in Mainland Southeast Asia and South China (Fig. 1a–d). The rapidly rising sea level flooded land bridges and reduced land area and consequently resulted in large-scale land use change, including forest reduction and fragmentation^7,10,11,45–47^. The rise in sea level and associated paleogeographic changes are therefore likely the key drivers for the population splits reported here, resulting in the population structure and genetic diversity found in present-day Southeast Asia.

Tibeto-Burmans also include a small portion of Malaysian Negrito ancestry as shown in the ADMIXTURE results (Fig. 2c) and are close to Southeast and East Asians in the PCA (Fig. 2b). This is likely because of their close relationships with East Asians^48^. The qpGraph modelling for Tibeto-Burmans shows Dravidians (33%) and East Asians (67%) as the best-fitted 2-way admixture ancestors (Supplementary Fig. 10, Supplementary Tables 4 and 5). Based on the MSMC estimates, the splits between Tibeto-Burmans and Mainland Southeast Asians/East Asians occurred relatively recently—~7000 and ~8000 years ago respectively (Supplementary Fig. 7 and Supplementary Table 3). MSMC-IM estimates show that the migration between Tibeto-Burmans and Mainland Southeast Asians/East Asians continued until ~3000 years ago (Supplementary Fig. 7d). Thus, the Malaysian Negrito ancestry found in Tibeto-Burmans is likely from the common ancestor with mainland Southeast Asians.

### Population size and density estimates

**Fig. 4:**
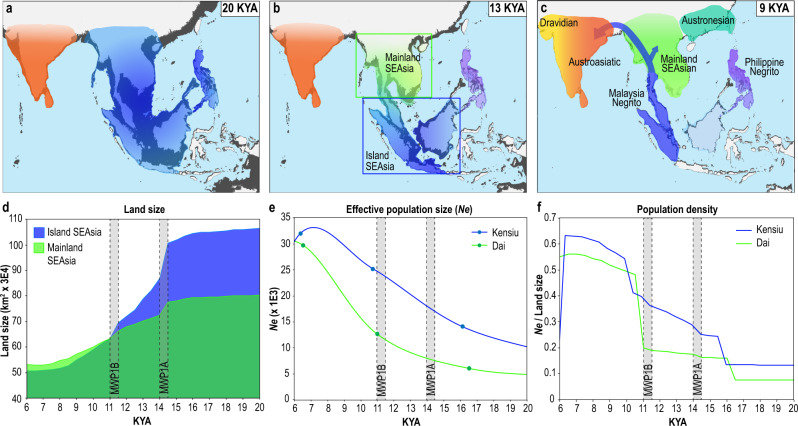
Land size changes and reconstructed demographic history. We hypothesised population migration and splits on the map along with the sea-level changes. The geographic regions inhabited by the ancestral populations are suggested based on the current locations of their descendants and represented as coloured shading in the map. The base maps were retrieved from the public dataset^83^ with open access. The changes in inhabitancies over time are reconstructed based on our demographic history estimates. **a** 20 thousand years ago (KYA), the ancestral population of Southeast Asians likely inhabited Sundaland. The ancestors of the Austroasiatic speakers in South Asia might have occupied the Indian subcontinent. **b** 13 KYA after MWP1A, the ancestral Southeast Asians split into three populations. **c** 9 KYA after MWP1B, a population split occurred between Mainland Southeast Asians and Austronesians. The blue arrows represent the admixture of the Malaysian Negritos with South Asians and Mainland Southeast Asians and do not represent the migration route. South Asians split into two or three populations around this time. **d** Estimated size of landmass of Island (blue) and Mainland Southeast Asia (green) since 20 KYA as their regions are indicated in **b**. **e** The fitted curves of MSMC estimates of effective population size (*N*_*e*_, *Y* axis) over time (KYA, *X* axis) for Kensiu and Dai populations, as representatives of Malaysian Negritos and Mainland Southeast Asians from Island and Mainland Southeast Asia, respectively. The estimate shown in this figure is one of the repeated runs (Supplementary Fig. 6). We selected the representative estimate as the result that is the closest to the midpoint among the results. **f** Estimated population density (*N*_*e*_ divided by land size, *Y* axis) over time (KYA, *X* axis), since 20 KYA for the two regions indicated in **b**. Compared to the land size, the time resolution of the MSMC estimates is smaller, and the sharp increases of the density around 16 and 11 KYA are shown. The numerical data for the plot. **d**, **e**, and **f** are shown in Supplementary Table 1d.

During the deglacial period, the estimated effective population size shows that Southeast Asian populations, except for Igorot, expanded four- to seven-fold by ~11,000–6000 years ago, relative to their population sizes during the LGM (Supplementary Fig. 6a). The effective population size of the Kensiu, who occupied Island Southeast Asia, was relatively large compared to the Dai from Mainland Southeast Asia over the deglacial period (Fig. 4e). In contrast, the land reduction in Island Southeast Asia was larger: 45% compared to 26% in Mainland Southeast Asia between 20,000 and 11,000 years ago (Fig. 4a–d, Supplementary Table 1). The population density (effective population size divided by land size) in Island Southeast Asia increased at least 8.6 times from the LGM and was larger than the density in Mainland Southeast Asia for most of the period between the LGM and mid-Holocene (Fig. 4f). The increase in population density of Island Southeast Asia was driven largely by population expansion (Fig. 4e), which occurred at the beginning of the Holocene around 11,000 years ago after MWP1B. This increase in population density in Island Southeast Asia likely drove the migration of Malaysian Negritos toward Mainland Southeast Asia and further on to South Asia (Fig. 4c).

## Discussion

Our interdisciplinary paleogeographical and whole genome-based approach advances the understanding of the impact of sea-level changes on human populations from previous studies^23–28^. For example, a recent study^28^ on Philippine populations suggested that the climate-driven changes after the LGM may have prompted population differentiation in Island Southeast Asia. They hypothesised the history of population migrations into the Philippines based on their estimation of population divergence or admixture using the genotyping dataset and related their results with the paleogeographic map. Our study extended the geographic regions and populations including Southeast Asia as well as South Asia to focus more on the impact of the paleogeographic changes on the people who lived in Sundaland during the rapid sea-level rises. Furthermore, we incorporated a 3D (laterally heterogeneous) glacial isostatic adjustment (GIA) model^31,32^, instead of a 1D (laterally homogeneous) GIA model^28^, to infer sea-level change histories, as 3D structure is essential and significantly important in GIA modelling^49^. Hence, we were able to infer both paleodemographic events relative to major changes in sea-level rises with the high-temporal resolution, as well as the concurrent human demographic history with fine resolution using high coverage whole genome sequence datasets.

We suggest that the two MWPs promoted multiple population splits (Supplementary Fig. 8). The Sundaland flooding during MWP1A and MWP1B reduced the land area and broke land bridges between Palawan, Borneo, Sumatra, and Malay Peninsula. The land reduction and splits also caused a reduction and fragmentation of forest cover^47^, and changes in the atmosphere-ocean dynamics, resulting in strengthening of the regional monsoon^50^. We show that after the population splits, the populations expanded (Fig. 4e, Supplementary Fig. 7) during a time of reduced rates of sea-level rise and favourable environmental conditions. The reduction of rates of sea-level rise since MWP1B stabilised coastlines^51^ (Fig. 1 and Supplementary Movie) and led to the formation of extensive coastal wetlands^52,53^ and peatlands^54^. Under these conditions, coastal margin productivity increased^55^, which in turn improved the availability of high-nutrition food for human consumption^56,57^. The surface temperature increased by ~7 °C since the LGM^58,59^ with the warming ocean and increasing atmospheric moisture leading to strengthening of the East Asian Monsoon^60,61^.

Our integrated analysis enabled us to estimate the population density during the rapid sea-level rises in Southeast Asia. The increasing population density reflects the pressures of environmental changes and could be a driving force for the migration of the Malaysian Negritos into South Asia (Fig. 4). The ancient admixture occurred in the earliest inhabitants of South Asia by back migration of the earliest settlers in Southeast Asia. Previous studies using genetic and linguistic analyses examined the potential admixture between the South and Southeast Austroasiatic speakers^27,39,48,62–64^. The conclusions were either unclear or contradictory on the origin of Austroasiatic speakers. One of the studies estimated that the origin of the Southeast Asian admixture found in South Asian Austroasiatic groups was Daic speakers^27^. Thus, we included the Dai population in the MSMC analysis as mainland Southeast Asians although the population was from Xishuangbanna in China, the area along the border of Burma and Laos. We show that this Dai population in our dataset has a similar composition of ancestries to most Thai populations by analysing them together with the comprehensive Thai population dataset^65^. The analysis results are shown in Supplementary Fig. 11.

In our study, we identified the origin of admixture as the Malaysian Negritos, not the Dai population. The MSMC-IM estimates support the hypothesis of gene flow between Kensiu (Malaysian Negritos) and Dai as well as between Kensiu and Birhor (South Asian Austroasiatic), but not between Birhor and Dai (Fig. 3c). In addition, the estimated proportion of gene flow based on qpGraph from Malaysian Negritos to the South Asian Austroasiatic group is larger (29%) than from Malaysian Negritos to Mainland Southeast Asians (3%). The admixture of Malaysian Negritos to South Asian Austroasiatic groups is likely to have been direct from Malaysian Negritos rather than through Mainland Southeast Asians who contain the Malaysian Negrito ancestry. Our novel finding of the origin of admixture was possible because of our extensive datasets, including the Negrito groups and fine resolution of analyses using whole genome datasets.

Importantly, the ancient gene flow of the Southeast Asian ancestries to South Asian Austroasiatic groups and the associated time estimates are only revealed by our analyses (Fig. 3c, Supplementary Fig. 7). The finding of admixture between Mainland Southeast Asians and Malaysian Negritos suggests a migration route of Malaysian Negrito groups along the Indochina western coastline, a journey we estimate to have taken 1.5 – 2 years assuming a two-hour daily walk. As such, the Malaysian Negritos may be the first casualties of sea-level rise, having lost 50% of the inhabitable territory since the LGM, after establishing their populations across Sundaland for the last ~70,000–50,000 years^11^. Unbeknown until now, their forced migration left a remarkable genetic footprint in South Asians, thereby contributing to the genetic makeup of one of the largest and growing human populations today.

## Methods

### Global mean sea level and paleotopography maps

We inferred the magnitude and rate of sea-level rise in 500-year increments between 26,000 years ago and the present, to map high-resolution temporal and spatial palaeogeography of Southeast Asia (Fig. 1 and Supplementary Movie). The maps are based on the ICE-6G_C global ice history model^2,30^ and HetM-LHL140 3D Earth model^31,32^, using the Sundaland Shelf^10,66^ and Singapore^33^ as example locations amongst a global far-field dataset^1^ (see Supplementary Table 1).

We reconstructed sea-level change from far-field sites that are distal to ice sheets at the LGM and constrain GMSL change including MWP-1A and 1B^1,4,10,60,66–73^.

In Southeast Asia, this includes sea-level index points (SLIPs) from sediment cores extracted from the now-submerged Sundaland Shelf region by ref. ^10,66^ (Fig. 1a). From these sediment cores, radiocarbon dating of sea-level indicators from intertidal depositional environments including mangrove deposits and mudflats constrain sea levels between the LGM and the Holocene. The SLIP data show a rise in sea level between 21,000 and 19,000 years ago from −116 to −114 m (Fig. 1b). Between 19,000 and 14,600 years ago, sea levels increased from −114 to −96 m before increasing rapidly from −96 to −80 m between 14,600 and 14,300 years ago. Between 14,300 and 13,100 years ago, sea level rose from −80 to −64 m. The Sundaland Shelf SLIP data are consistent with a global compilation of SLIPs from other far-field sites in ref. ^1^ (Fig. 1a, b). Here, the age uncertainties are incorporated into the elevational range uncertainty of the sea-level data (further details of this calculation can be found in Supplementary Table 1 of ref. ^1^). The GMSL prediction from the ICE-6G_C model^2,30^ fit the SLIP data within the uncertainty range and show a rise in GMSL of 135 m since 26,000 years ago. Between 14,500 and 14,000 years ago (MWP1A), GMSL rose from −93.8 to −70.9 m at a rate of 46 mm/yr and between 11,500 and 11,000 years ago (MWP1B), GMSL rose from −43.6 to −32.7 m at a rate of 22 mm/yr (Fig. 1b).

Studies of relative sea-level (RSL) change around the Sundaland Shelf region have provided further constraints to the regional sea-level history in Southeast Asia since the early Holocene^33,74–79^. In Singapore, SLIPs are based primarily on mangrove peats^33,76,77^ and have been standardised following protocols in the compilation of RSL databases^80^. The SLIPs show RSL rose from −21 m to −0.7 m between 9500 and 7000 years ago (Fig. 1b). Sea levels continued to rise to a mid-Holocene highstand of 4 m at 5200 years ago before falling to the present thereafter driven by hydro-isostatic processes.

We reconstructed the RSL history and generated paleotopography maps following Peltier 1994^81^ and 2004^82^:

$$T\left(\theta ,\,\lambda ,\,t\right)=S\left(\theta ,\,\lambda ,\,t\right)+\left[{T}_{p}\left(\theta ,\,\lambda \right)-S\left(\theta ,\,\lambda ,\,{t}_{p}\right)\right]$$

Here, $\theta ,\,\lambda ,$ and $t$ represent latitude, longitude and time, respectively; $T\left(\theta ,\,\lambda ,{t}\right)$ is the paleotopography at time $t$; ${T}_{p}\left(\theta ,\,\lambda \right)$ is the present-day topography from ETOPO1^83^, $S(\theta ,\,\lambda ,\,{t}_{p})$ and $S\left(\theta ,\,\lambda ,{t}\right)$ are the present-day sea level and sea level at time $t$ respectively, which are predicted by a GIA model with the ICE-6G_C ice history model^2,30^ and the HetM-LHL140 3D earth model^31,32^. The ICE-6G_C model has been tuned to fit RSL data and GPS data in North America, Eurasia, and Antarctica. The ICE-6G_C model prediction of RSL in other far-field regions including Barbados fits well with the high-quality deglacial RSL records. The HetM-LHL140 3D earth model includes lateral variations both in the lithospheric thickness and mantle viscosity. We generated paleotopography maps every 500 years from 26,000 years ago to the present (Fig. 1c–e, Supplementary Movie) from which we calculated land cover change compared to the land cover at the LGM as a percentage land loss (Fig. 1c inset).

### Meltwater pulses (MWP1A and MWP1B)

Superimposed on long-term secular GMSL rise following the LGM delegation have been rapid short-term increases in sea level, termed meltwater pulses (MWPs)^4^. Fairbanks^4^ first identified MWPs during deglacial sea level using radiocarbon-dated cores of coral reef crest species *Acropora palmata* in Barbados. refs. ^67,71^ subsequently confirmed and validated their existence in Barbados corals using U/Th ages. Increases in sea level during MWPs result from the rapid influx of meltwater to the ocean as Late Quaternary ice sheets decayed, and ice-dammed and subglacial lakes drained in response to global climate amelioration. The rapid rise in sea level during MWP1A has been associated with the melting of the Northern Hemisphere ice sheets including the Laurentide, Cordilleran, and Eurasian Sea ice sheets^1,5,6,73,84^. Further evidence to support MWP1A at the global scale has also been documented in Tahiti^67,85,86^, Papua New Guinea^87,88^, Sundaland Shelf^10,66^, and the Great Barrier Reef^89^ among other locations^6,90^.

A second rapid rise in sea level (MWP1B) identified in Barbados coral records^4,71,72,91^ is also supported by evidence in the Western Pacific^92,93^ and polar Arctic^93^. Rising sea levels during MWP1B have been associated with the melting of the Laurentide, Innuitian, and West Antarctica ice sheets^2,6,72^. The significance of MWP1B at the global scale, however, has been contested due to the lack of an equivalent magnitude of change recorded in the Tahiti record^67,94^. Carlson and Clark^90^ and Lambeck et al.^1^ further contest evidence for MWP1B while ref. ^94^ discussed the potential influences of subduction zone tectonics in Barbados and the reliability of *Acropora palmata* as an accurate sea-level indicator. While MWPs are typically viewed as a global phenomenon, it has become evident that their timing and spatial extent show regional and local variability as more geological records have become available^6^. Nonetheless, new evidence to support MWP1B from revised Barbados coral records suggests sea levels rose 8–11 m in ~250 years beginning at ~11,300 years ago^95^.

### Whole genome sequencing data

We used the genome datasets generated and provided by the GenomeAsia 100 K consortium^29^ and selected 763 whole genomes, representing 59 ethnic groups, from the GenomeAsia 100 K pilot datasets (Fig. 2a and Supplementary Table 2). The data are available from the European Genome-phenome Archive (EGA) under accession number EGAS00001002921. The procedures of sample collection, ethical approvals for the studies, genome sequencing, and variant calling have been described previously^29^. The total number of variants included in the variant call dataset is 33,047,521 biallelic Single Nucleotide Polymorphisms (SNPs).

### Population structure and admixture analyses

We removed SNPs with >2% missing rate, minor allele frequency <0.01, and linkage disequilibrium *r*^2^ > 0.2 from the dataset and analysed the remaining 1,141,813 SNPs, using PLINK 1.9^96^. Individual ancestries were estimated using ADMIXTURE v1.3.0^35^, which implements a block relaxation method to calculate ancestry fractions from allele frequencies. We applied ADMIXTURE with *K* = 4–20 to the dataset with 10 repeated runs using different seeds (Supplementary Fig. 3). The consistency of the results was calculated using CLUMPP1.1.2^97^. PLINK 1.9^96^ was used to perform a Principal Component Analysis (PCA)^34^ using “-pca” option (Supplementary Fig. 2).

We performed the ADMIXTURE^35^ analysis on the X chromosome of the dataset with the option of -haploid = “female:23” (Supplementary Fig. 3). The frequencies of the Y chromosome and mitochondrial haplogroups were calculated in each group of the Indo-European and South Asian Austroasiatic groups and the Kensiu (Supplementary Fig. 5). The haplotype identification was retrieved from a previous study^29^.

### Population history modelling

The inbreeding coefficient (*F*) and Runs of Homozygosity (ROH) for every individual were calculated with the 1,141,813 SNPs filtered as the same as in the admixture analysis using PLINK 1.9^96^ (Supplementary Fig. 4). ROH calculation was performed with the default parameters of PLINK.

For the modelling of population phylogenetic relationships using Treemix^40^ and qpGraph^41^, we excluded outlier individuals based on the *F* and admixture analysis. Among the 11 population groups we categorised, some inconsistent or admixed ethnic groups compared to other groups within each population group were excluded, and in total we used 630 individuals for Treemix and qpGraph analyses. The rooted phylogenetic tree for the 11 population groups without migration event was constructed by Treemix with 100 iterations with window size of 1000 and 2000 SNPs. The outgroup was given as Andamanese. The consensus tree among the 100 trees with bootstrap values was identified by PHYLIP-Phylogeny^98^ (Supplementary Fig. 9). Both consensus trees were used for the given topology for the qpGraph modelling for the 2-way admixture events for three population groups, South Asian Austroasiatic groups, Mainland Southeast Asians, Tibeto Burmans, as admixed populations and tested all pairs of 10 population groups (Supplementary Tables 4 and 5). We also modelled the 3-way admixture events with the given topology of Supplementary Fig. 9b (Supplementary Table 6). For the main figure (Fig. 3a), we show the modelling results used the given topology with window size 2000 SNPs, based on the bootstrap values (Supplementary Fig. 9b). The detailed procedure and results are described in Supplementary Figs. 9, 10, and Supplementary Tables 3, 4, 5, and 6.

### Effective population size and population split estimation

To infer population sizes, effective population sizes, and coalescence rates among the studied populations, we analysed representative ethnic groups from each population with the Multiple Sequentially Markovian Coalescent (MSMC) software^42,43^. Based on the ADMIXTURE and PCA results, we selected eight to ten non- or less- admixed individuals for each of the representative ethnic groups: the Paniya (Dravidians), Birhor (South Asian Austroasiatic groups), Gujjar (Indo-Europeans), Kensiu (Malaysian Negritos), Aeta (Philippine Negritos), Igorot (Austronesians, *n* = 2), and Dai (Mainland Southeast Asians). We estimated population size changes using four haplotypes (two individual genomes) for each population and estimated the population split between a pair of populations using eight haplotypes (two individual genomes from each pair of populations) using MSMC2^42,43^. For each ethnic group and each pair of groups, we performed four to five runs using different sets of individuals to assess the robustness of estimates (Supplementary Fig. 6).

The outputs of MSMC2 were applied to MSMC-IM^44^ to fit the Isolation-migration (IM) model to infer the migration rate over time for a pair of populations. Our population split and migration rate estimations are based on the MSMC-IM results. Population split times were decided based on the MSMC-IM estimates at the coalescence rate = 0.5 as suggested in the previous study^42,43^. We performed four to five independent runs to estimate the time and present them as a range for the population split time. Each run used a different pair of individuals (Supplementary Figs. 7, 8, and Supplementary Table 3).

Both time estimates of population size changes and splits were in the number of generations and scaled by a mutation rate of 1.25 × 10^−8^/site/generation and a generation time of 29 years, following ref. ^99^.

Phasing data were used from a genome dataset generated in a previous study^29^. We excluded chromosome 6 from the MSMC analyses due to possible phasing errors in the HLA gene cluster region.

### Reporting summary

Further information on research design is available in the Nature Portfolio Reporting Summary linked to this article.

## Supplementary information

Supplementary Information

Description of Additional Supplementary Files

Supplementary Data

Supplementary Movie

Reporting Summary

## Data Availability

The datasets used in this study are available in the EGA under accession number EGAS00001002921.
