# Supplementary material for: Prehistoric human migration between Sundaland and South Asia was driven by sea-level rise
Source: Commun Biol. 2023 Feb 4;6:150. doi: 10.1038/s42003-023-04510-0 (PMC9899273; doi:10.1038/s42003-023-04510-0)
Supplement: Supplementary file 1 — Supplementary Information [file 42003_2023_4510_MOESM1_ESM.pdf]

Supplementary Information for

Prehistoric human migration between Sundaland and South Asia was driven by sea-level rise

Hie Lim Kim, Tanghua Li, Namrata Kalsi, Hung Tran The Nguyen, Timothy A. Shaw, Khai C. Ang, Keith C. Cheng, Aakrosh Ratan, W. Richard Peltier, Dhrubajyoti Samanta, Mahesh Pratapneni, Stephan C. Schuster, Benjamin P. Horton

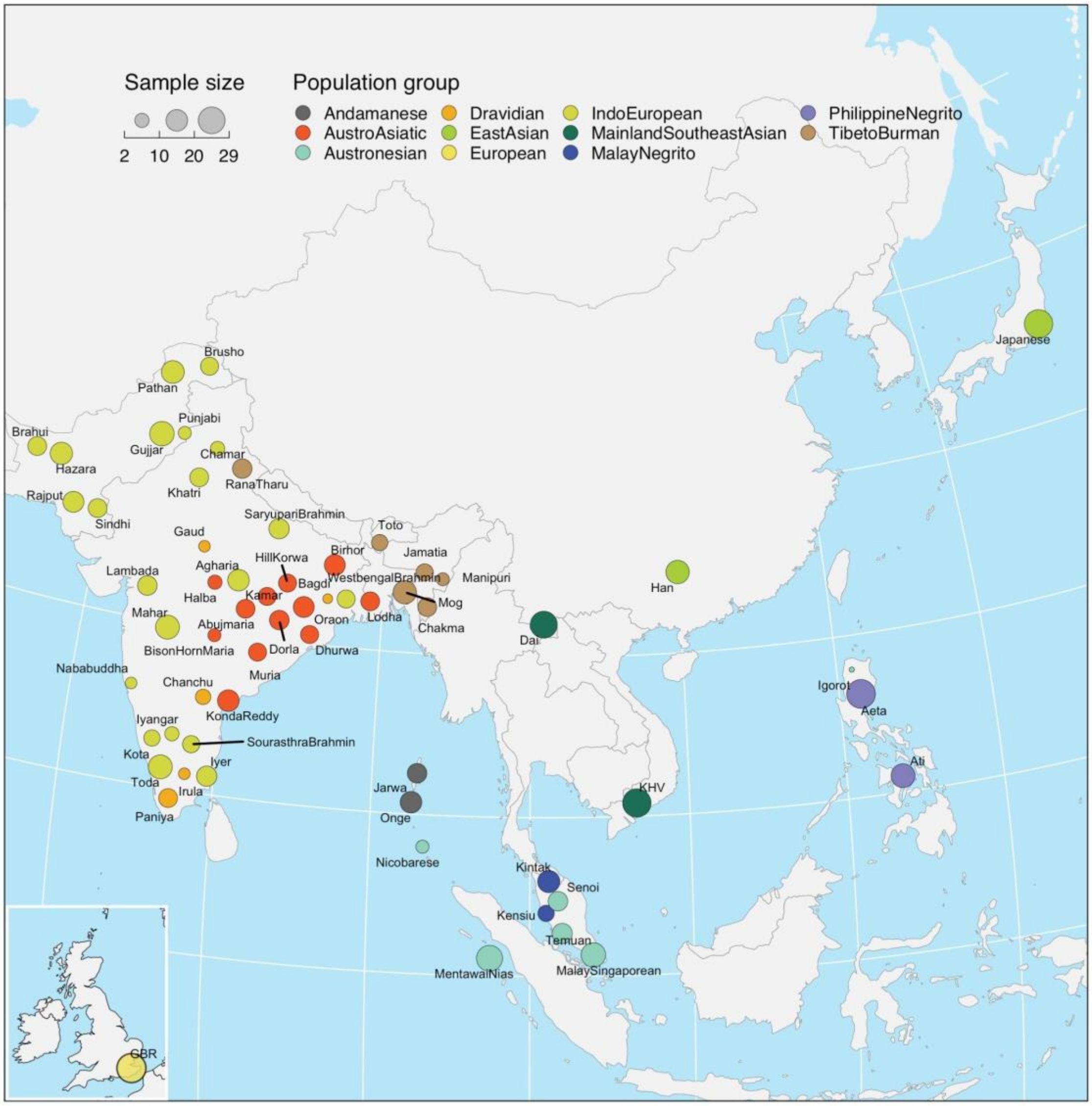

Supplementary Figure 1. Sample distribution map. The figure below shows the location of the populations in the geological map except for the GBR. The sample distribution in the map is the same figure in Figure 2A in the main text. This figure includes the population label.

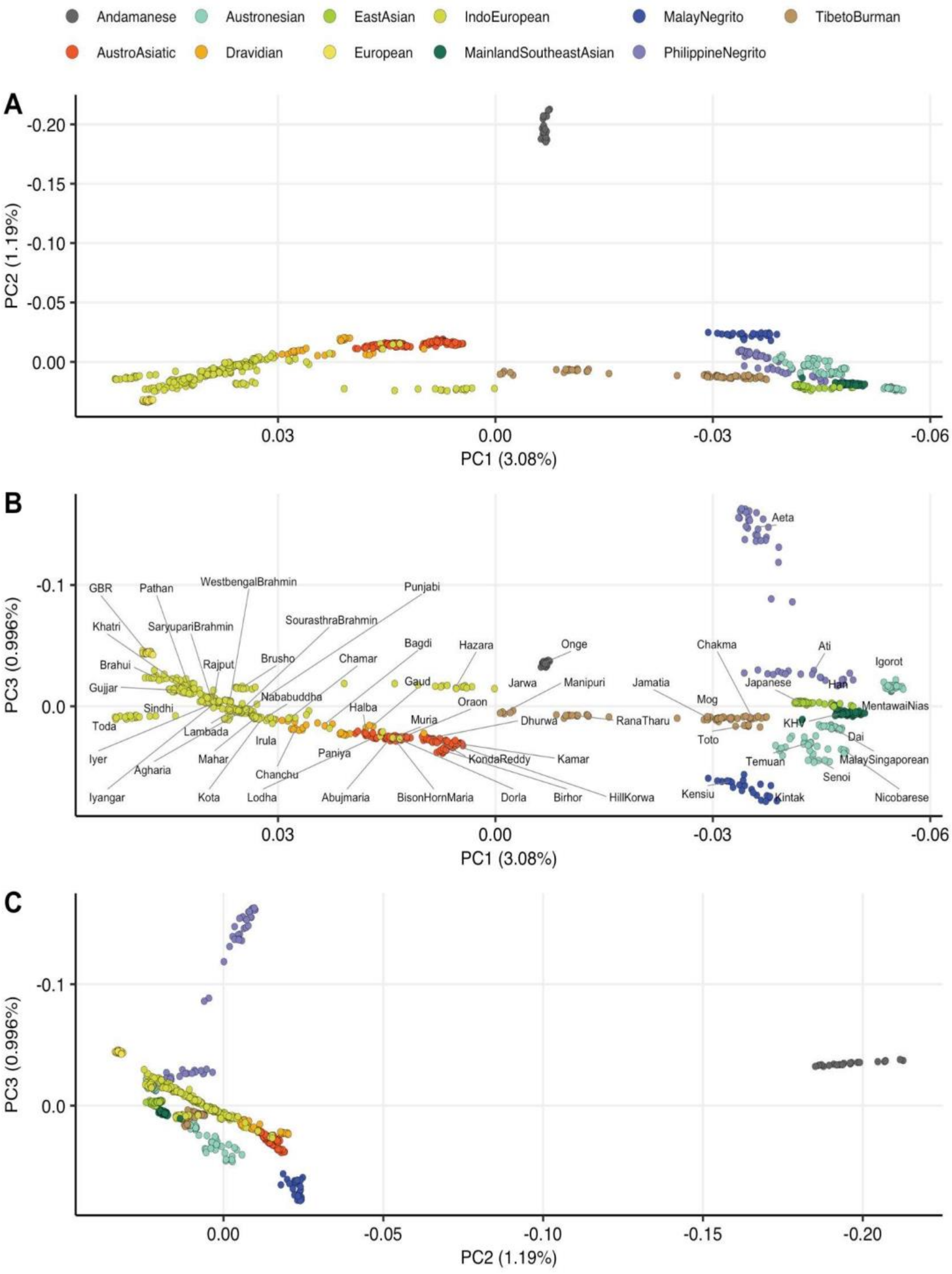

Supplementary Figure 2. PCA plots with ethnicity label. **A** the same plot is shown in Figure 2C in the main text. **B** and **C** PCA plots calculated for the same dataset plotted by PC1 vs. PC3 and PC2 vs. PC3, respectively. Plot **A** and **B** are very similar to each other because it is largely determined

by PC1 (22.8%) which represent the difference between South and Southeast Asians. Plot **C** is different because either PC1 and PC2 represents each of South and Southeast Asians.

A

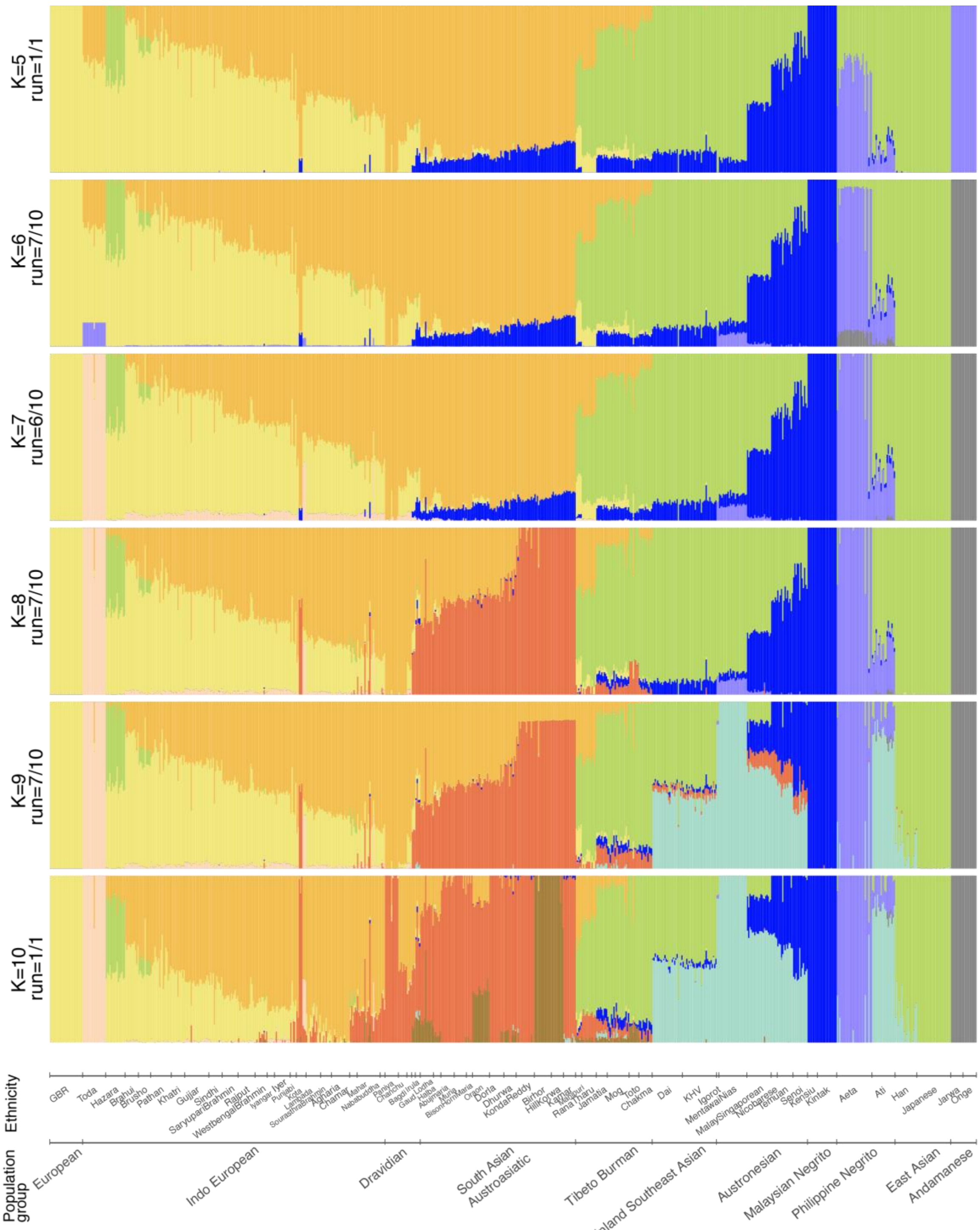
K=5
run=1/1
K=6
run=7/10
K=7
run=6/10
K=8
run=7/10
K=9
run=7/10
K=10
run=1/1
Ethnicity
Population group
European
Indo European
Dravidian
South Asian Austroasiatic
Tibeto Burman
Mainland Southeast Asian
Austronesian
Malaysian Negrito
Philippine Negrito
East Asian
Andamanese

B

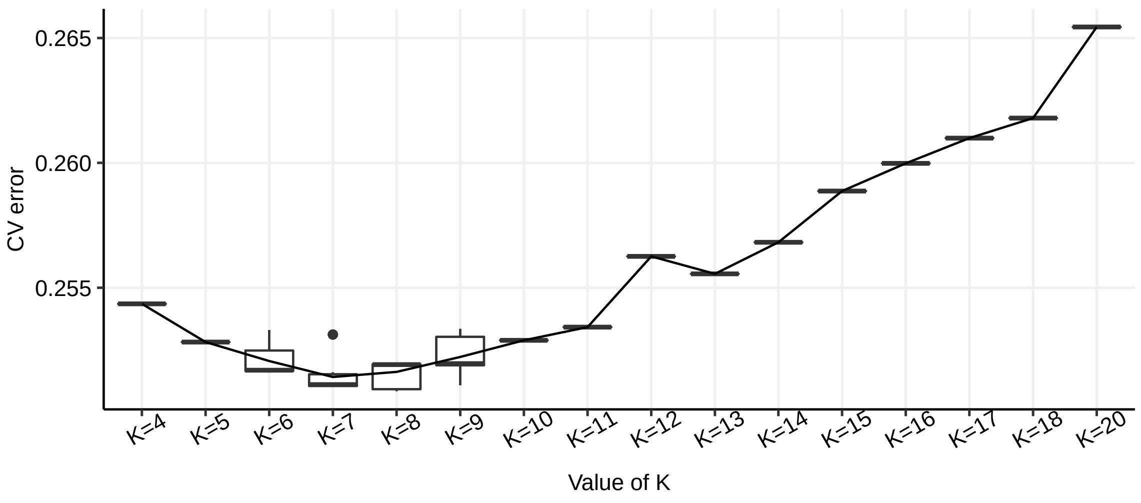

0.265
0.260
0.255
CV error
K=4
K=5
K=6
K=7
K=8
K=9
K=10
K=11
K=12
K=13
K=14
K=15
K=16
K=17
K=18
K=20
Value of K

C

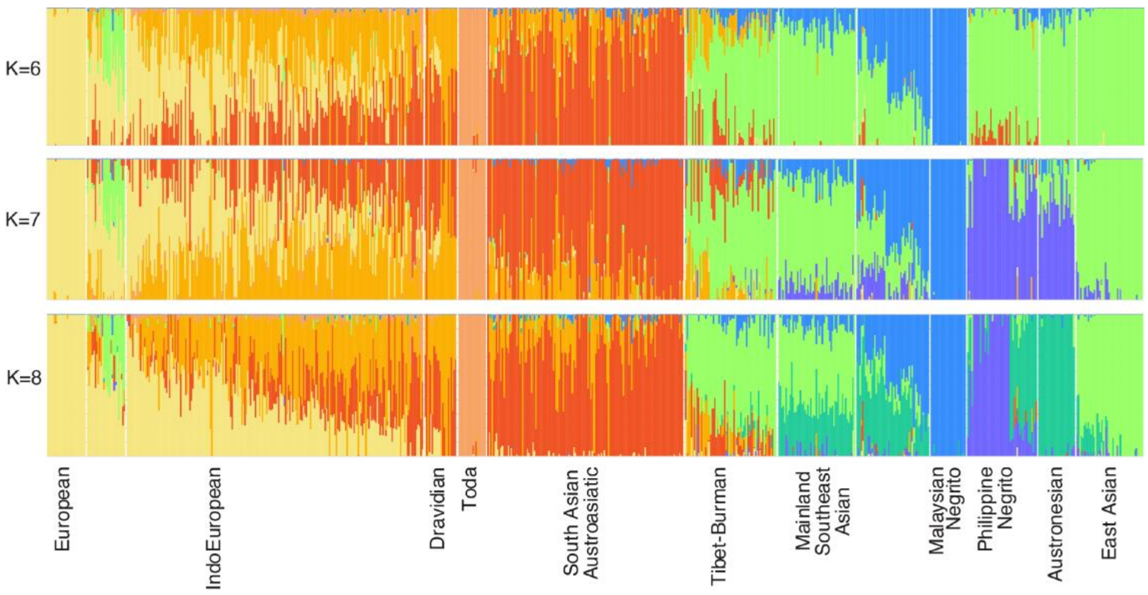

K=6
K=7
K=8
European
IndoEuropean
Dravidian
Toda
South Asian Austroasiatic
Tibet-Burman
Mainland Southeast Asian
Malaysian Negrito
Philippine Negrito
Austronesian
East Asian

D

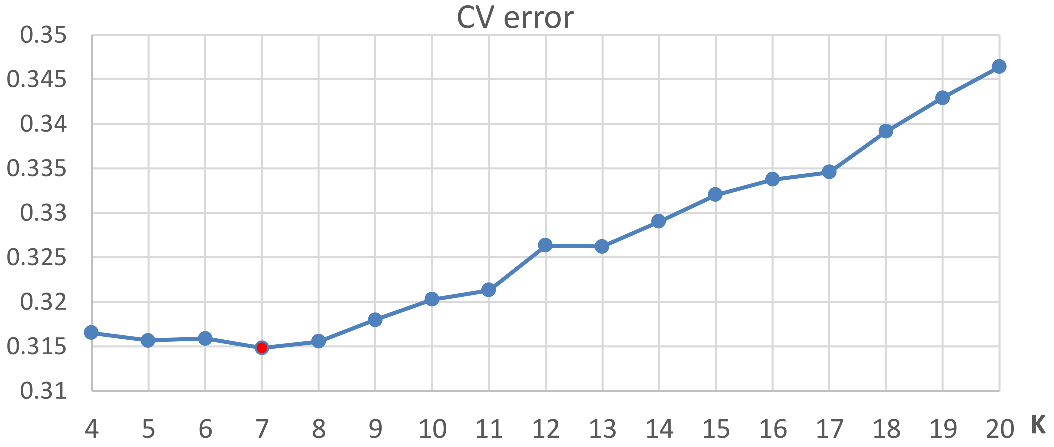

E

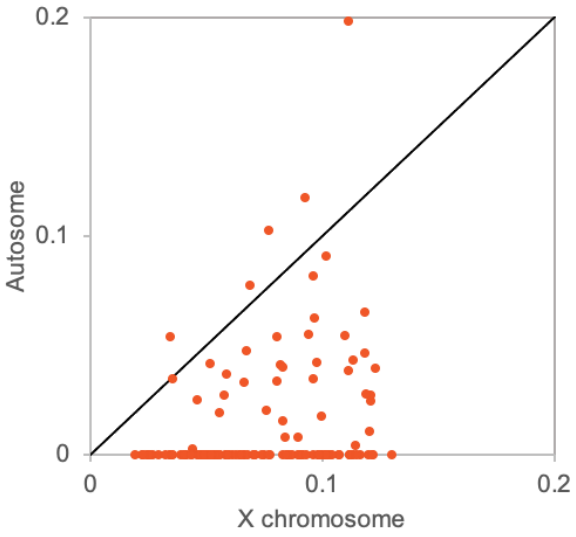

Supplementary Figure 3. Ancestral population structures in Southeast and South Asians are examined.

A Admixture plot for the 763 genome dataset. The proportions of ancestries of the 763 individuals are estimated for K=4-20 using ADMIXTURE1 and are shown in the plot for K=5-10. The main Figure 2c is the same plot of K=6-9 of this figure. In this figure, each population is labelled on the X axis.

B Cross-validation (CV) errors (Y axis) of K=4-20 (X axis) analyses are plotted. The optimal K is 7 based on the error rates, and the rates keep increasing with the higher Ks.

C Admixture plots of X chromosomes for the same dataset except for Andamanese. While the Malaysian Negrito ancestry (purple) in Indian Austroasiatic groups is present significantly in the

autosome data, the ancestry in the X chromosome is not significantly present. It suggests that Malaysian Negrito women had a little or minimum role to play in the admixture with Indian Austroasiatic populations. For the admixture with Mainland Southeast Asian groups, the Malaysian Negrito ancestry component appears clearly. Thus, the admixture events in India and Southeast Asia occurred in a different context.

D Cross-validation (CV) errors (Y axis) of K=4-20 (X axis) analyses for the plot **A**. The optimal K is 7 (red) based on the error rates, and the rates keep increasing with the higher Ks.

E Proportion of the Malaysian Negrito ancestry in South Asian Austroasiatic populations between autosomes (Supplementary Figure 3A) and X chromosome at K=7. The less contribution of X chromosome of Malaysian Negritos in South Asian Austroasiatic suggests male-driven migration.

A. **Runs of Homozygosity (ROH)**

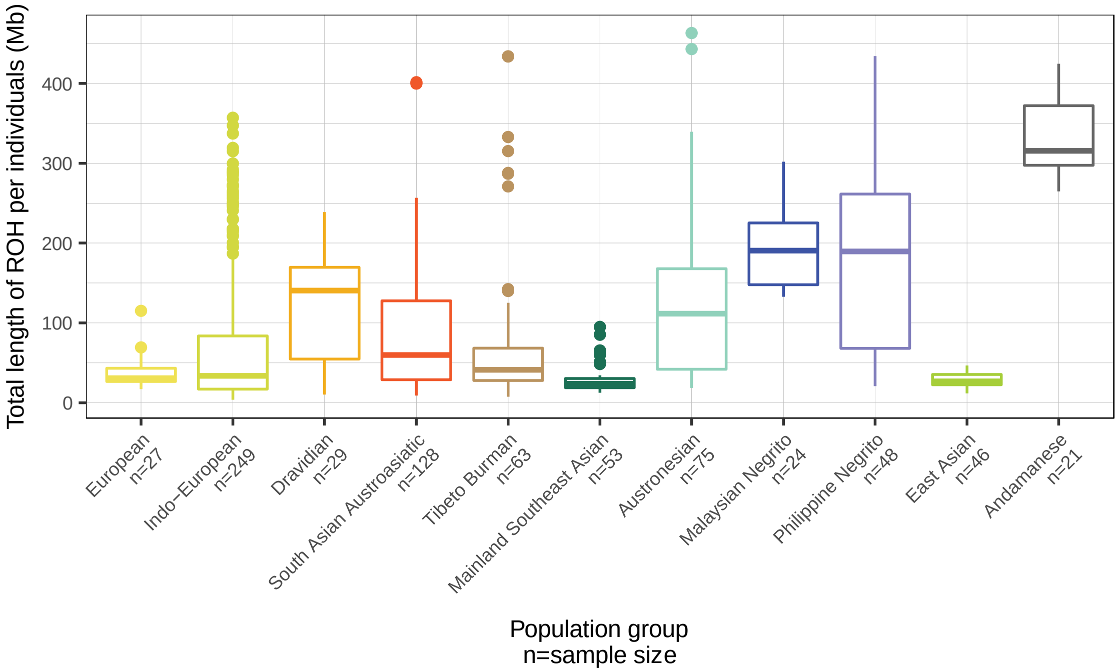

B. **Inbreeding coefficient (F)**

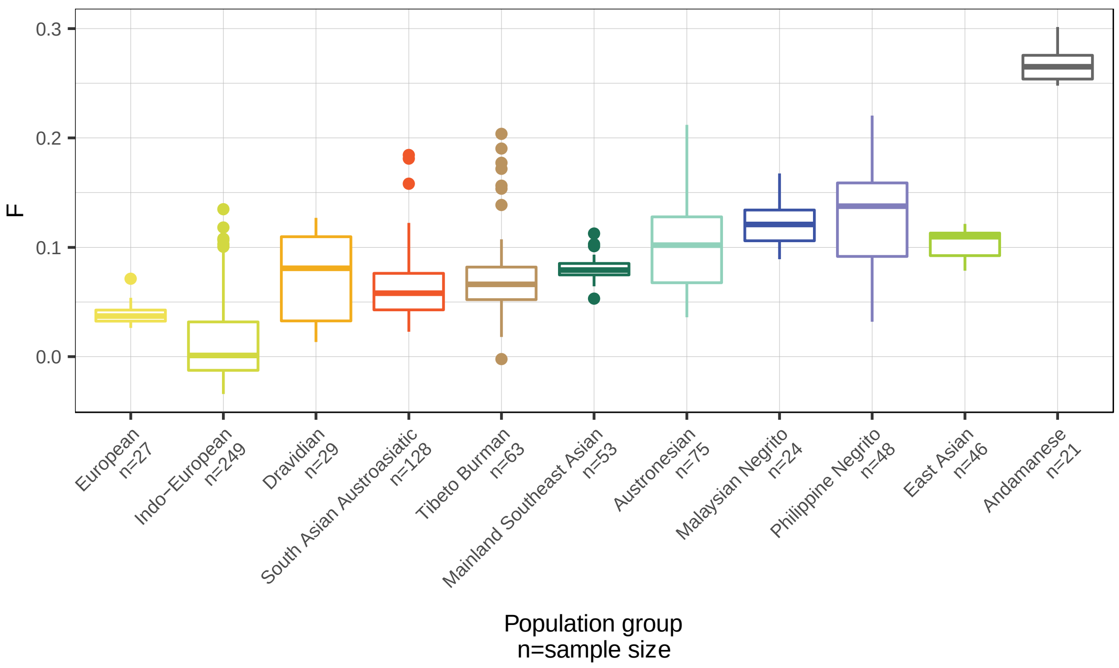

Supplementary Figure 4. Distribution of homogeneity of population groups

A Total length of Runs of Homozygosity (ROH) and **B** Inbreeding coefficient (F) for every individual were calculated with the 1,141,813 SNPs filtered as same as the admixture analysis using PLINK 1.92. A box plot for each parameter is shown for each of 11 population groups. ROH calculation was performed with the default parameters of PLINK: contain at least 100 SNPs, length of at least 1000kb; density of at least 1 SNP / 50kb on average; gap between 2 consecutive SNP less than 1000kb; scanning window contains 50 SNP and can tolerate 1 heterozygous call and 5 missing calls; SNPs must have hit rate of at least 0.05 on all scanning windows including it to be counted in the ROH.

F is calculated with the plink option –het. It estimates the excess of homozygotes compared to heterozygotes. Based on this value, we selected outliers for Treemix and qpGraph analyses since it provides a more conservative estimate of population group outliers and requires less assumptions of parameters compared to ROH.

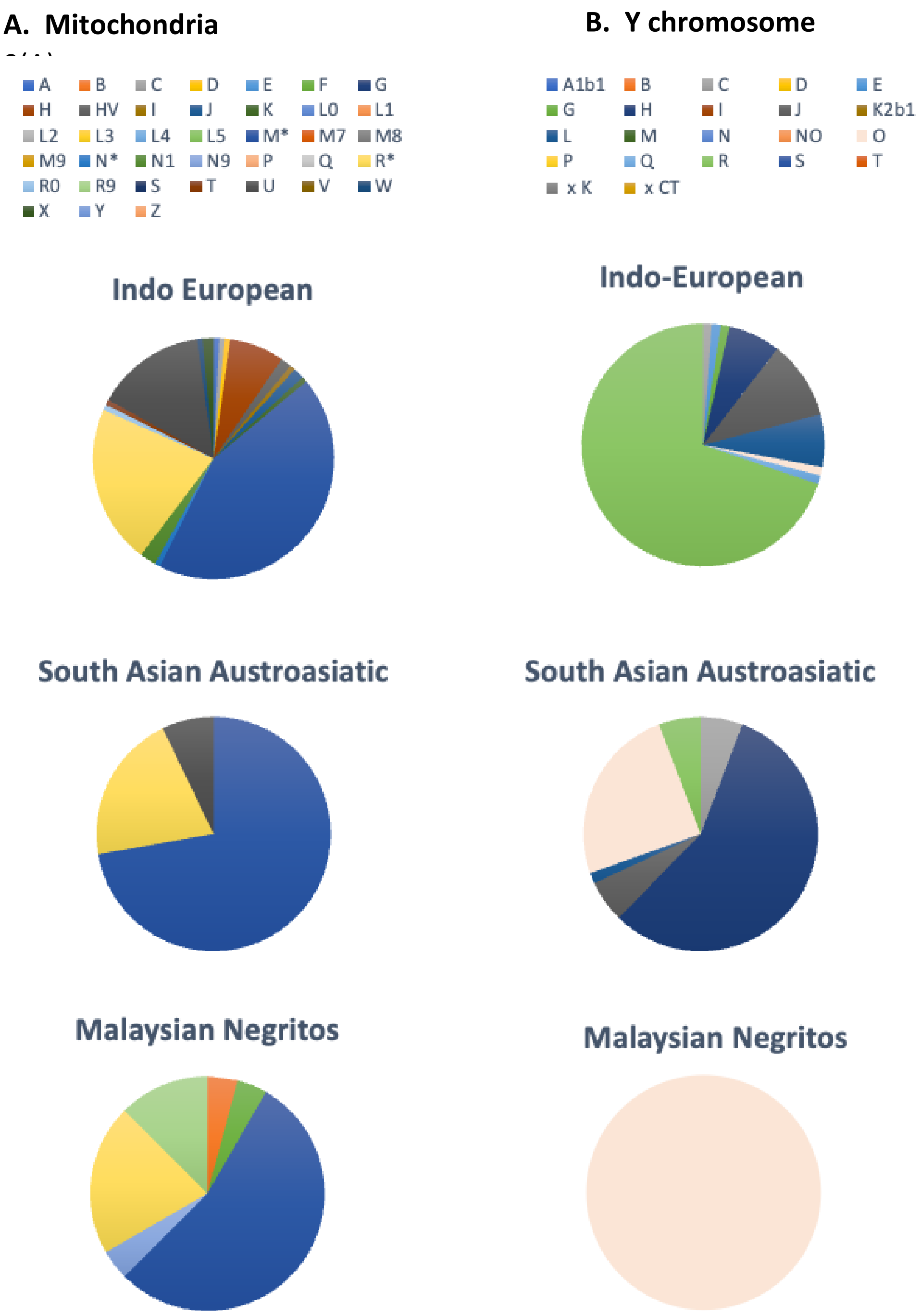

Supplementary Figure 5. Mitochondrial DNA and Y chromosomal haplogroup frequency. The haplogroup identification for mitochondrial DNA and Y chromosomes is retrieved from the previous study[3]. Frequencies of **A** mitochondrial and **B** Y chromosome haplogroups in Indo-European and South Asian Austroasiatic groups, and the Malaysian Negritos are shown in the pie chart. The South Asian Austroasiatic populations show a high frequency of the Y haplogroup “O”, which is found predominantly in Southeast Asians. No such signal is in the mitochondrial haplogroups.

A. Southeast and East Asia

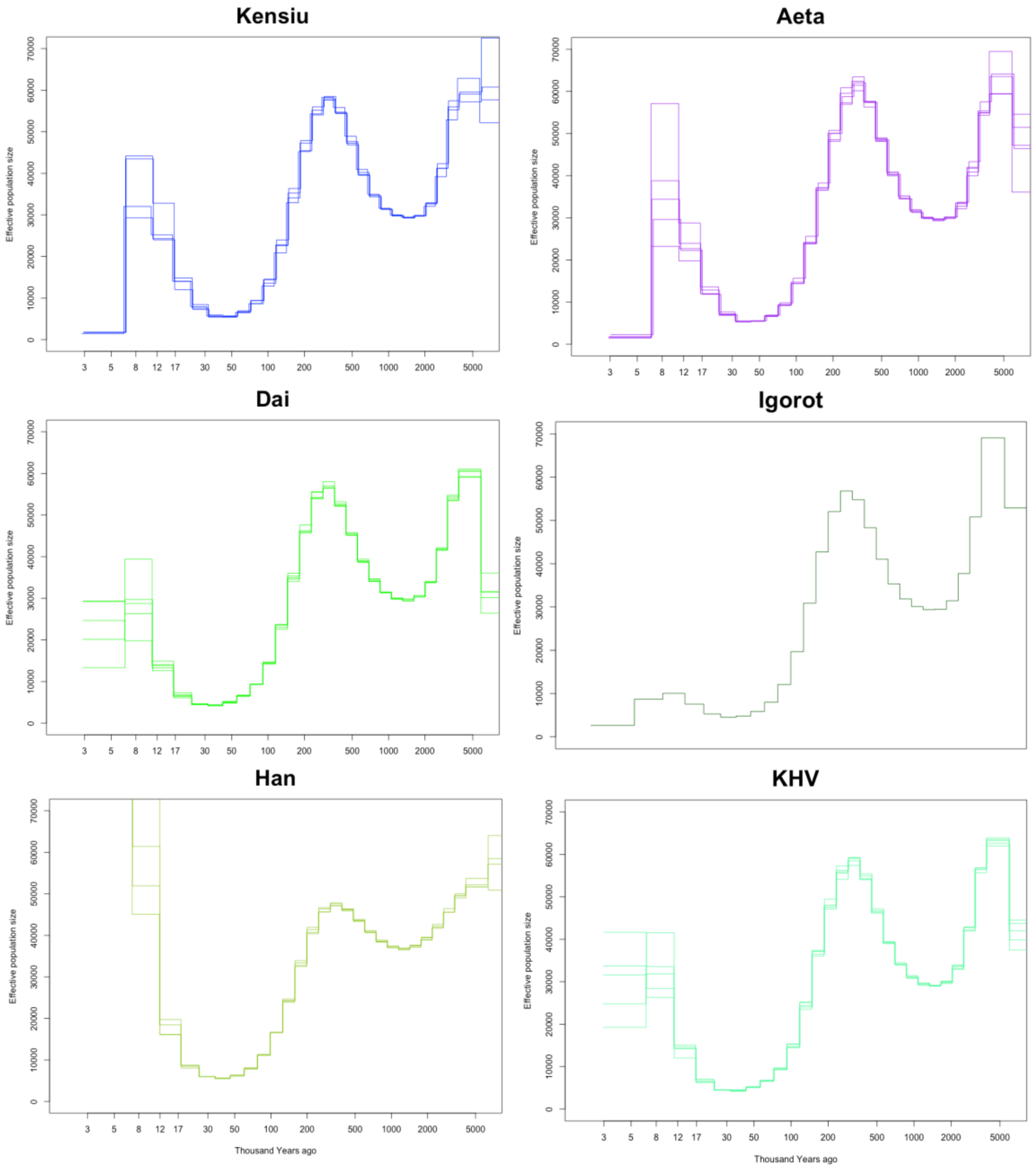

B. South Asia and European

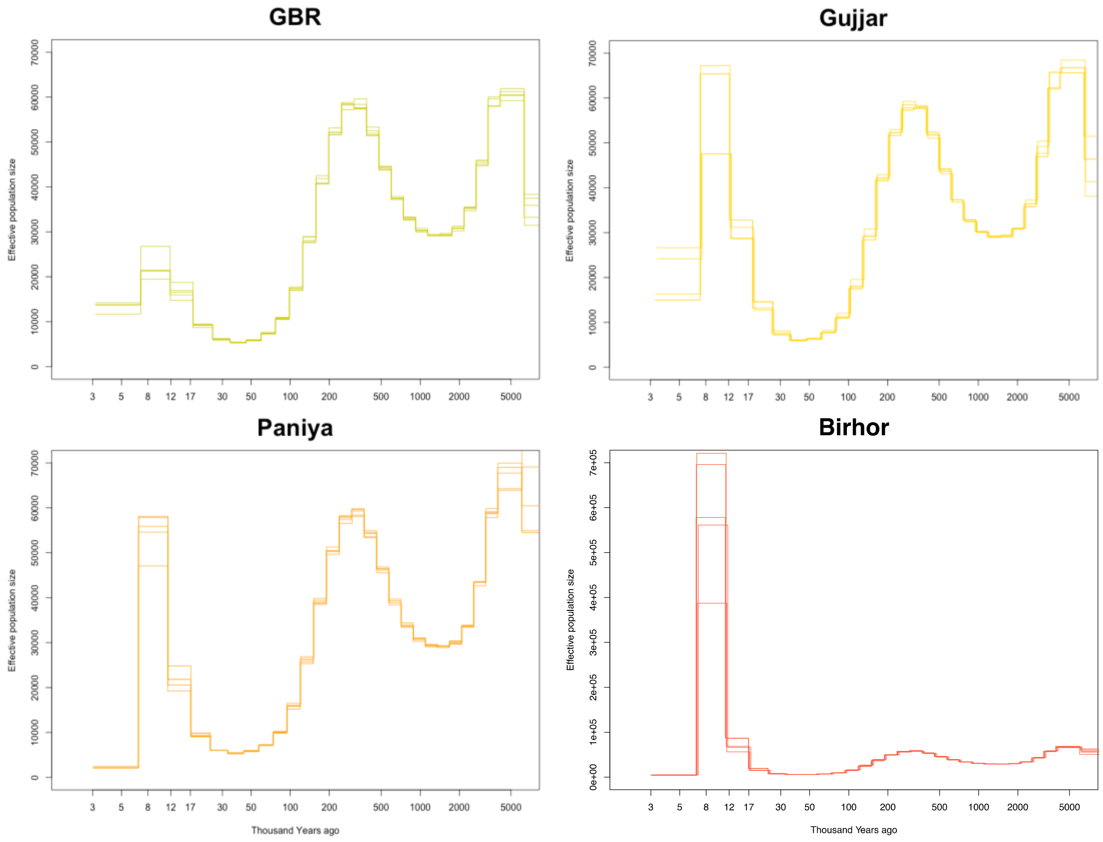

C. South Asian Austroasiatic groups

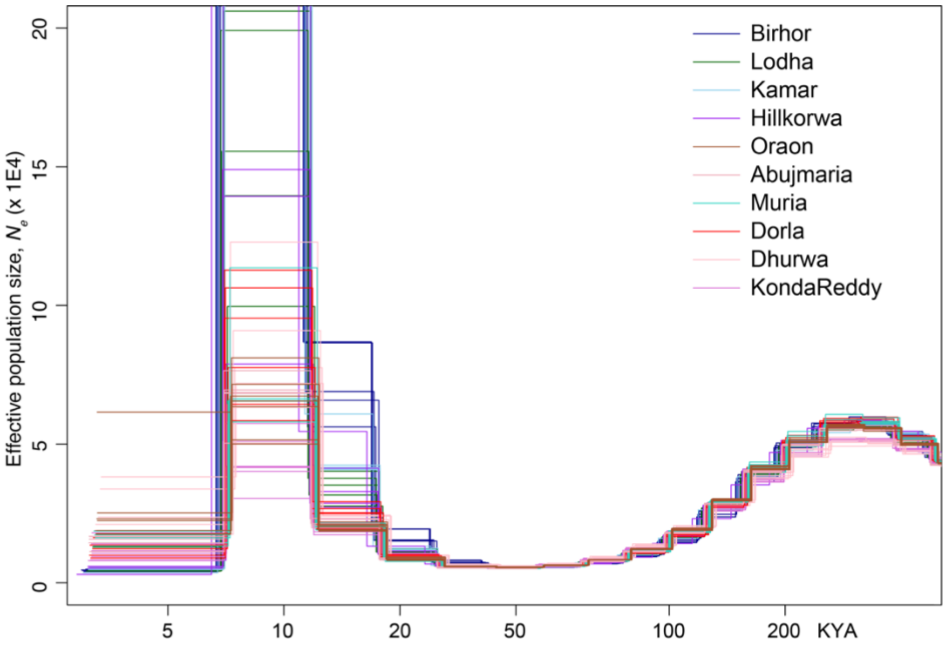

Supplementary Figure 6. Effective population size changes over time estimated by MSMC2[4,5]. One line indicates population size estimates from two individual genomes (four haplotypes), and every line shows estimates from different sets of individuals. For each population, we run two to five different sets of individuals and each result is plotted. The Y axis is the effective population size, and the X axis is log scaled time with a unit of thousand years ago (KYA). **A** shows MSMC estimates for the six Southeast and East Asian populations and **B** shows one European (GBR) and three South Asian populations. Only Birhor has a different scale for the Y axis because its population size is extremely high. **C** shows MSMC estimates for the 10 different ethnic groups of South Asian Austroasiatic populations. Many of the populations' effective population sizes are out the range of the Y axis and not shown in the plot.

A Southeast and East Asia

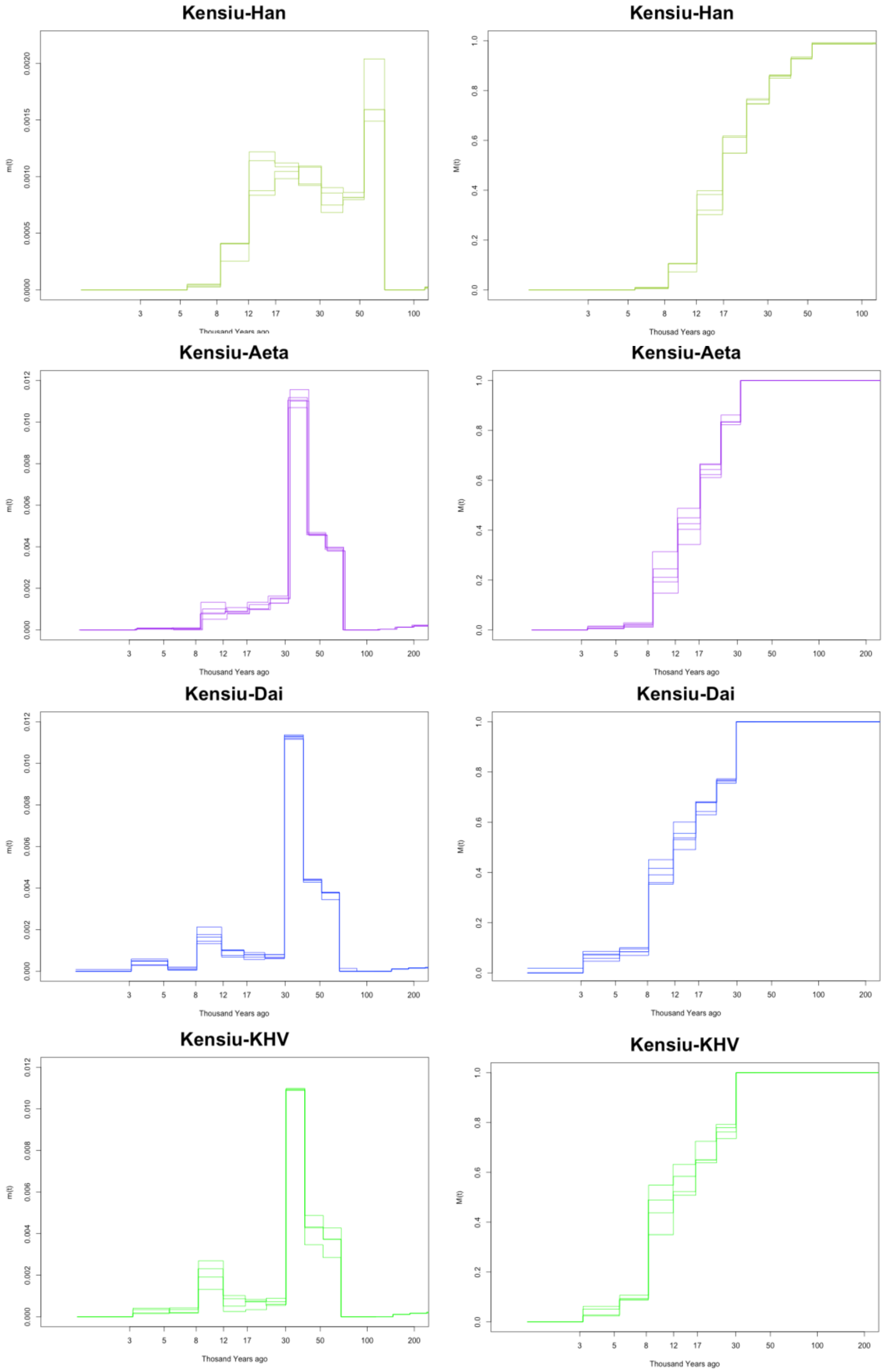

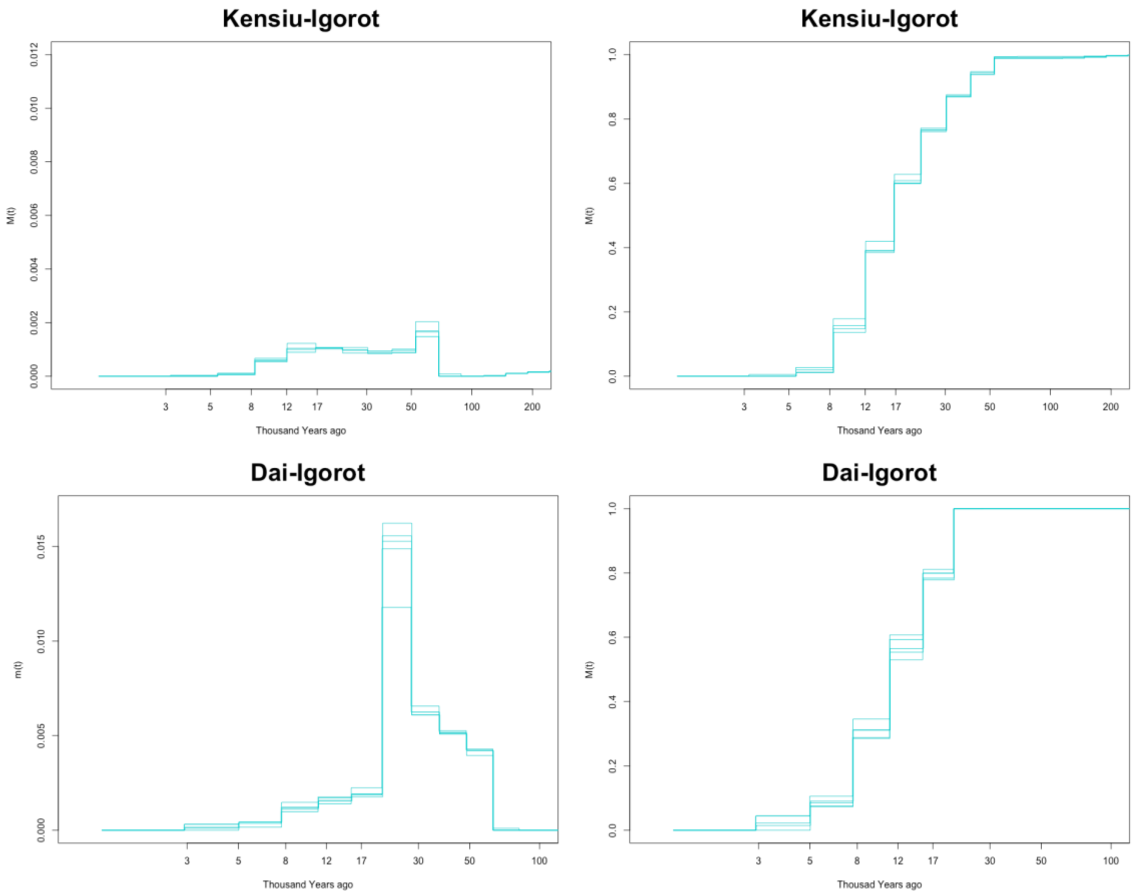
Kensiu-Igorot
M(t)
0.000
0.002
0.004
0.006
0.008
0.010
0.012
3
5
8
12
17
30
50
100
200
Thousand Years ago
Kensiu-Igorot
M(t)
0.0
0.2
0.4
0.6
0.8
1.0
3
5
8
12
17
30
50
100
200
Thosand Years ago
Dai-Igorot
m(t)
0.000
0.005
0.010
0.015
3
5
8
12
17
30
50
100
Thousand Years ago
Dai-Igorot
M(t)
0.0
0.2
0.4
0.6
0.8
1.0
3
5
8
12
17
30
50
100
Thousad Years ago

B South Asia

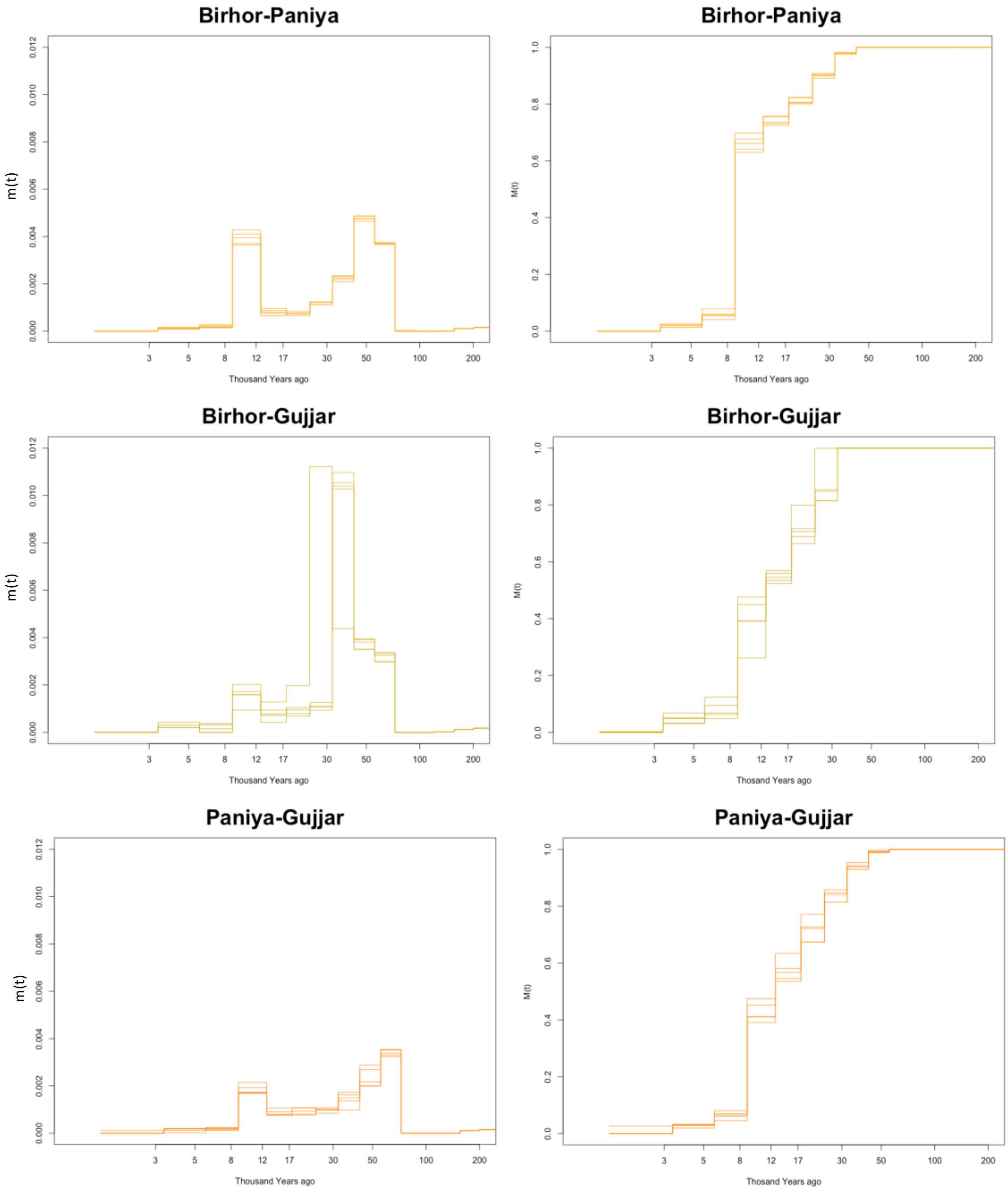

Birhor-Paniya
m(t)
Thousand Years ago
Birhor-Paniya
M(t)
Thosand Years ago
Birhor-Gujjar
m(t)
Thousand Years ago
Birhor-Gujjar
M(t)
Thosand Years ago
Paniya-Gujjar
m(t)
Thousand Years ago
Paniya-Gujjar
M(t)
Thosand Years ago

C Between Birhor and Southeast Asia

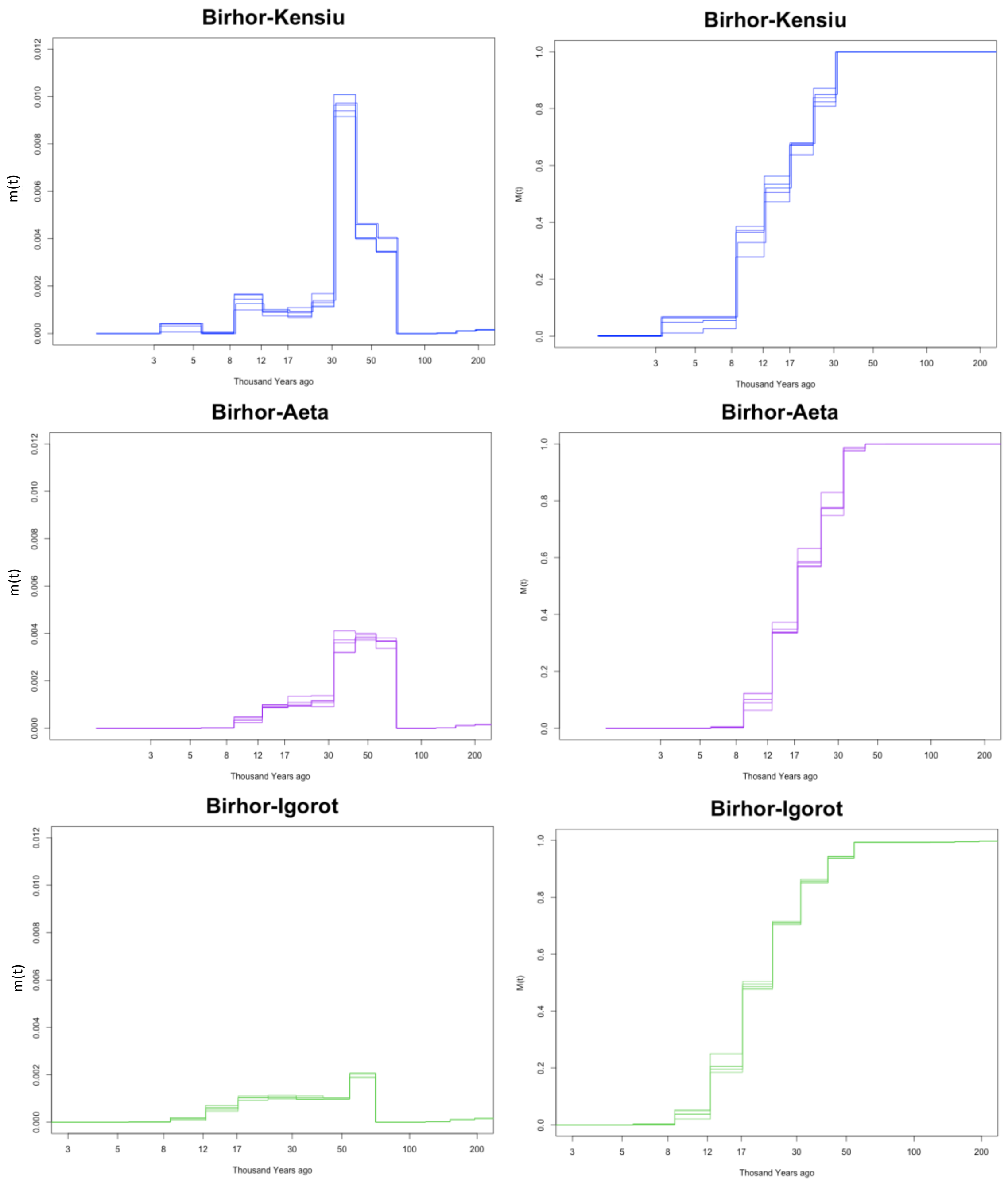

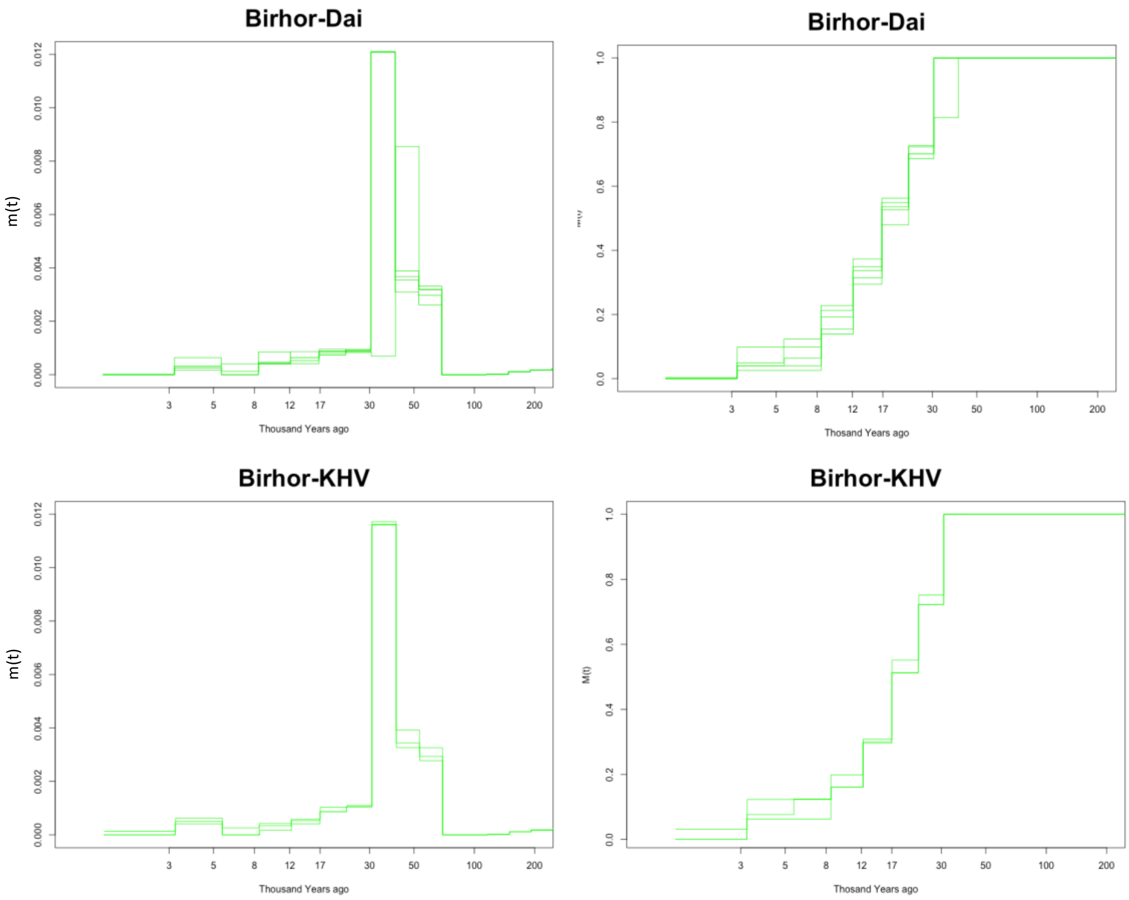
Birhor-Dai
m(t)
0.000
0.002
0.004
0.006
0.008
0.010
0.012
3
5
8
12
17
30
50
100
200
Thousand Years ago
Birhor-Dai
0.0
0.2
0.4
0.6
0.8
1.0
Thosand Years ago
Birhor-KHV
m(t)
Thousand Years ago
Birhor-KHV
M(t)
Thosand Years ago

D Between Tibeto-Burman and Others

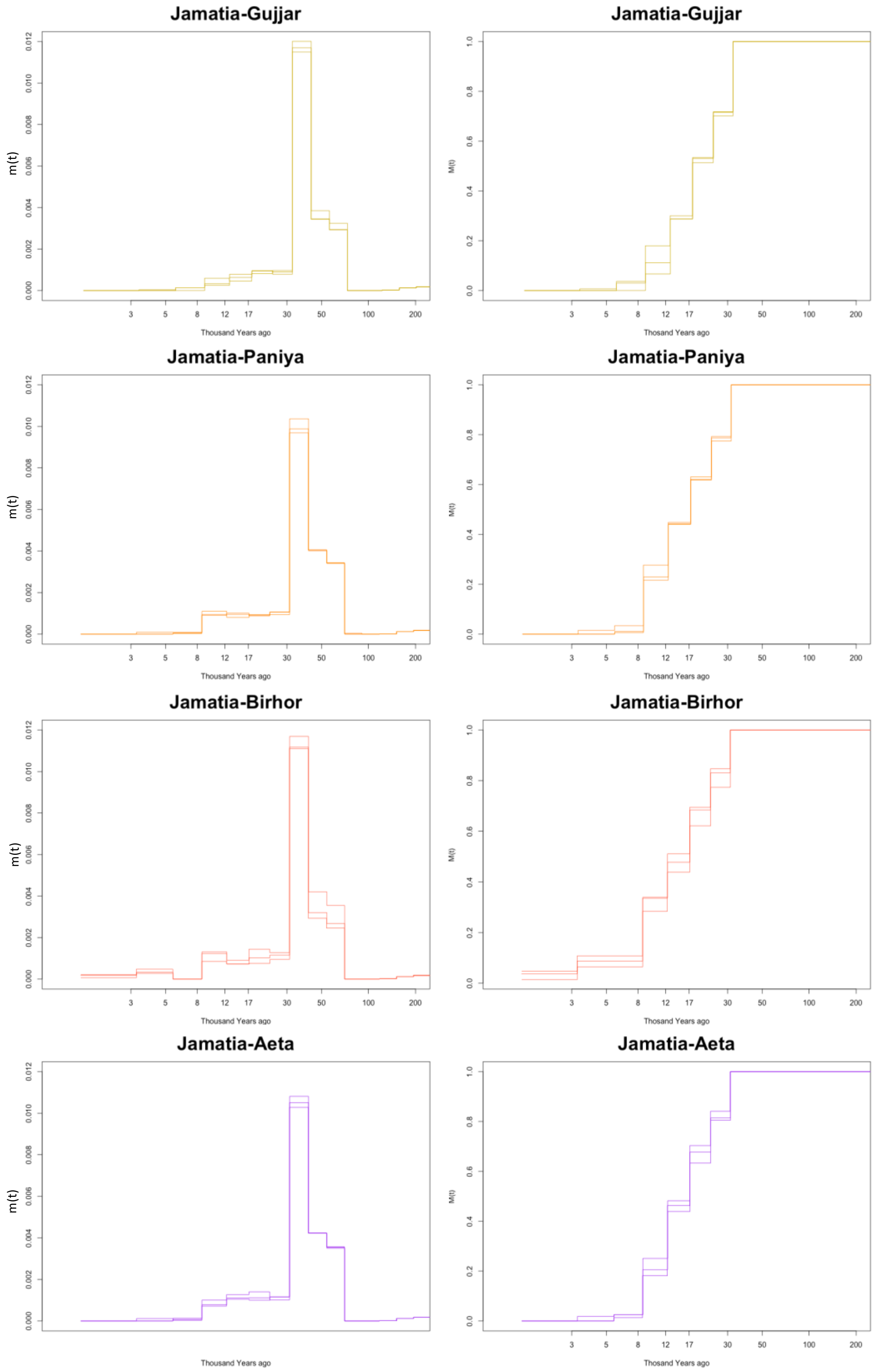

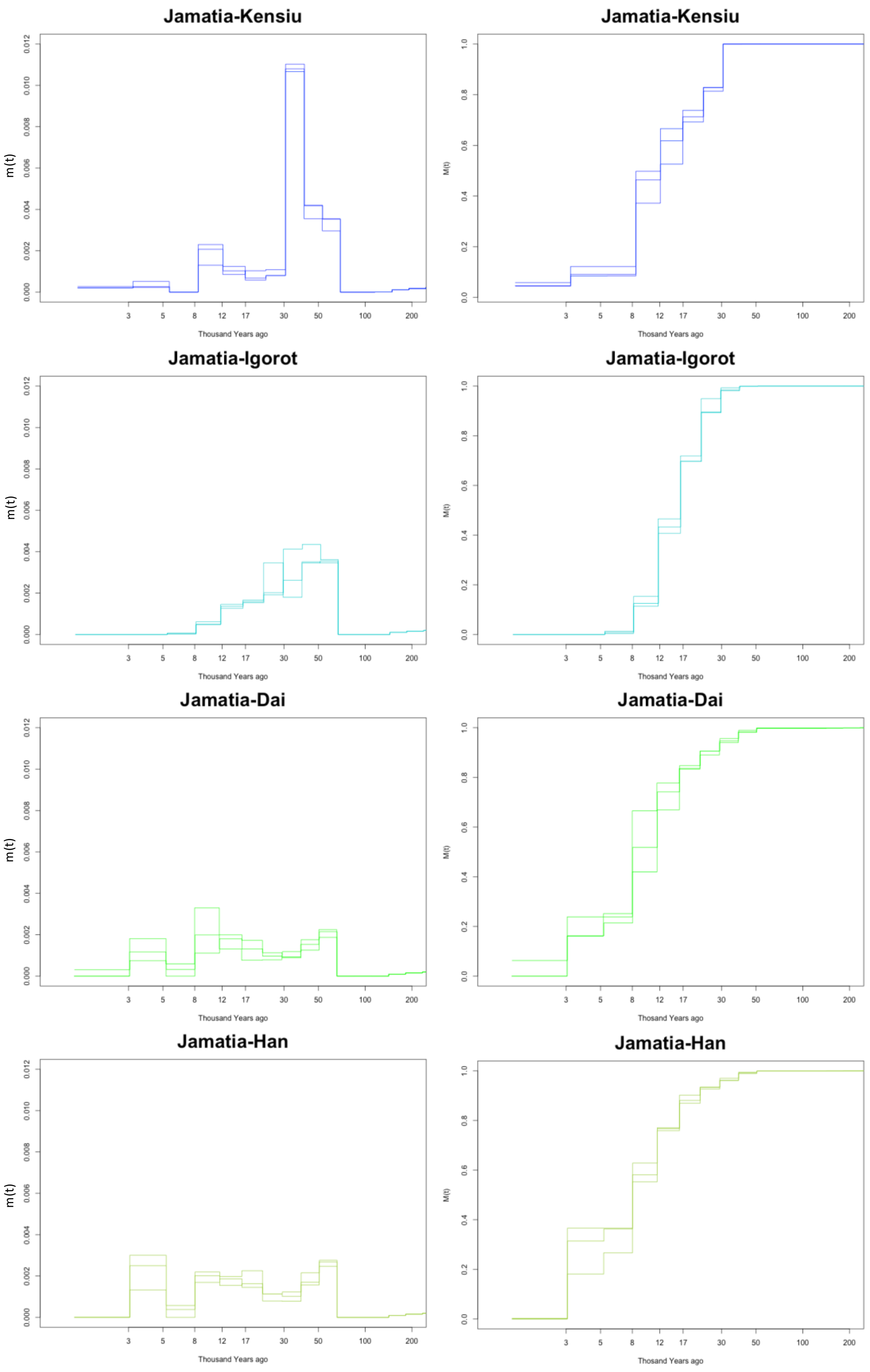

Jamatia-Kensiu
Jamatia-Kensiu
Jamatia-Igorot
Jamatia-Igorot
Jamatia-Dai
Jamatia-Dai
Jamatia-Han
Jamatia-Han
m(t)
M(t)
0.000 0.002 0.004 0.006 0.008 0.010 0.012
0.0 0.2 0.4 0.6 0.8 1.0
3 5 8 12 17 30 50 100 200
Thousand Years ago
Thosand Years ago

Supplementary Figure 7. Migration rate between populations over time estimated by MSMC-IM[6].

Population split times (years ago) for a pair of populations are estimated using MSMC-IM. The plots of left column show the migration rate between the two populations, m(t) on the Y axis over time *t* on the X axis, thousand years ago. On the plots of right column, the Y axis, *M(t),* is the cumulative migration probability that denotes the probability for lineages to have merged by the time *t*. The X axis is the time *t*, thousand years ago. One line indicates an estimate based on four individuals, two genomes from two different populations. For each plot there are three to five estimates from different combinations of individuals.

A pairs of Southeast and East Asian populations. The pairs Kensiu-Dai and Kensiu-KHV show very similar results, indicating the close relationships between Dai and KHV as mainland Southeast Asians. And only two pairs show the increase in the migration rate after population split about the same time, 8~12 thousand years ago. **B** pairs of South Asian populations. **C** pairs between Birhor and Southeast Asians. Only Kensiu shows the increase in the migration rate after split with Birhor. **D** pairs between Jamatia and other Asians. Jamatia (Tibeto-Burman) has split with East Asian (Han) most recently with continuous migration until ~3,000 years ago, as well as with Dai. Jamatia has the migration rate rise after split with Kensiu as same as Birhor-Dai.

A Within Southeast Asia

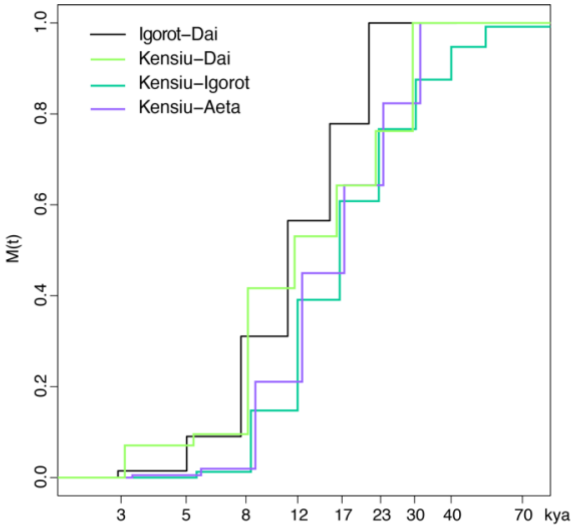

B Between Birhor and Southeast Asia

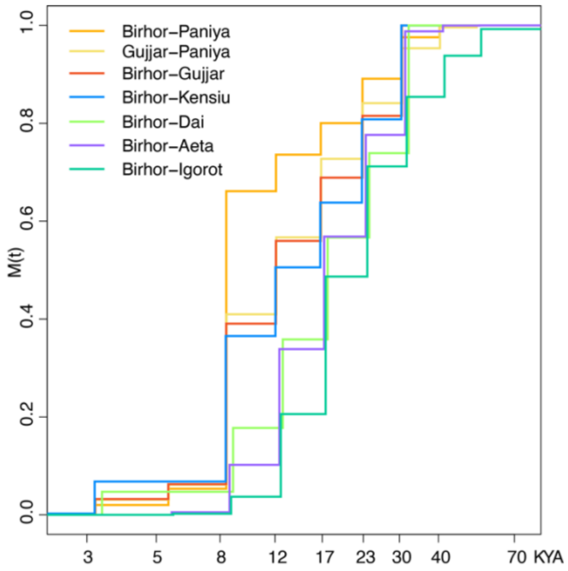

C Between Konda Reddy and Southeast Asia

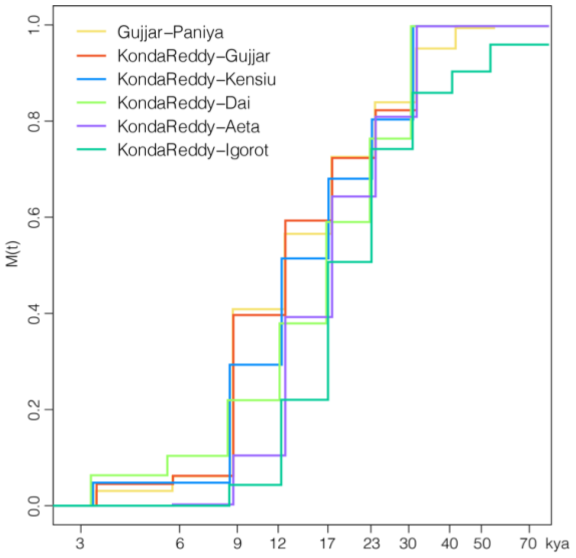

Supplementary Figure 8. Comparison of Population splits across different pairs of populations. Population split times (years ago) for a pair of populations are estimated using MSMC-IM[6]. The Y axis, *M(t),* is the cumulative migration probability that denotes the probability for lineages to have merged by the time *t*. The X axis is the time *t*, thousand years ago (KYA). The detailed results are shown in Supplementary Table 3.

A. Within Southeast Asian populations, the population splits of Kensiu-Aeta (blue) and Kensiu-Igorot (cyan) are overlapped, suggesting the population split across Malaysian Negritos, Philippine Negritos, and the common ancestor of Austronesians (Igorot) and mainland Southeast Asia (Dai) occurred in the close time frame period. The split between Igorot and Dai (black)

occurred more recently. The split between Kensiu and Dai (light green) is shifted to recent since about M*(t)*=0.5 compared to the splits of Kensiu-Aeta and Kensiu-Igorot, probably due to the admixture between Malaysian Negritos (Kensiu) and Mainland Southeast Asians (Dai) occurred around the time as we described in the main text.

B and C. The population splits between Birhor and Southeast Asian populations (B) and between Konda Reddy and Southeast Asian populations (C) are estimated and plotted. The results are similar between the two results, (B) and (C), suggesting the robustness of the estimates. The splits of Birhor with Dai (light green), Aeta (green), and Igorot (cyan) are the oldest and occurred in a similar period. The splits within South Asians (red, yellow, and orange), are younger than the splits between South Asians and Southeast Asians. Only the split of Birhor-Kensiu (blue) is more recent than the splits with other Southeast Asians. This is again likely a result of admixture between Malaysian Negritos (Kensiu) and South Asian Austroasiatic groups (Birhor and Konda Reddy).

A. The consensus tree from 100 iterations of Treemix runs (block size=1000 SNPs)

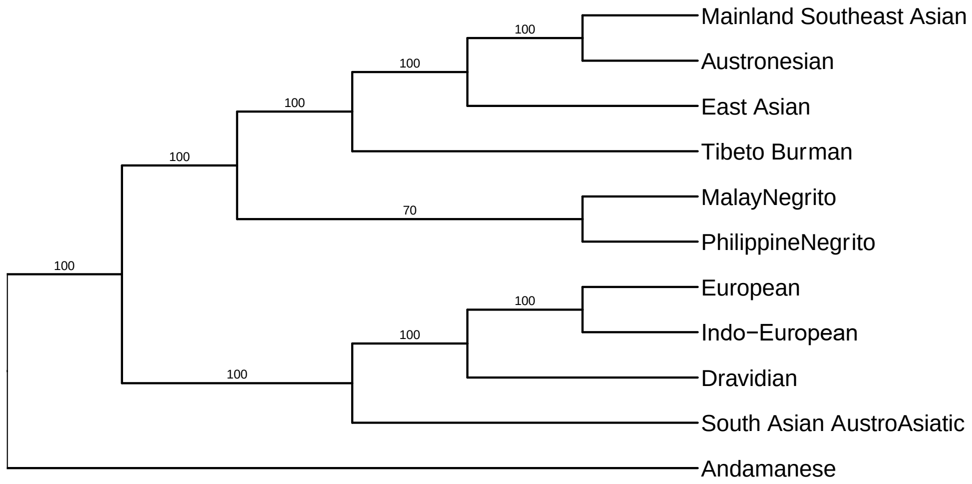

B. The consensus tree from 100 iterations of Treemix runs (block size=2000 SNPs)

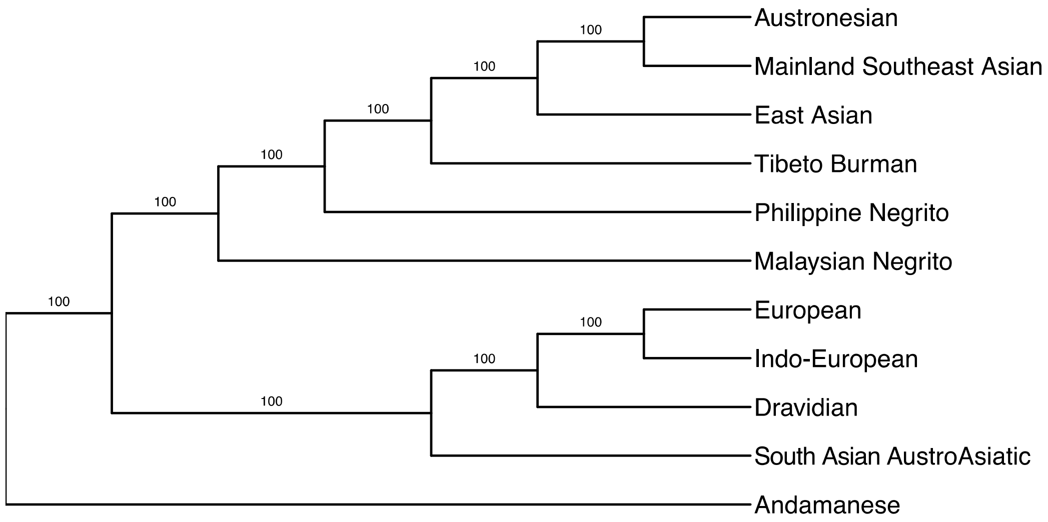

Supplementary Figure 9. Treemix analysis

To reconstruct population tree, we selected 630 individuals from 11 population groups after removing outliers based on degree of admixture and excess of homozygotes. In total 919,892 SNPs were used for performing Treemix[7] and Phylip-consense[8] analyses. Treemix was run with various size of a block (10 to 10,000 SNPs) and 100 iterations for bootstrapping the dataset. The root is determined with the given outgroup, Andamanese. Phylip-consense identifies a consensus tree among the iterations. The tree topology is same with the block size between 10 to 1000 SNPs (**A**), and the topology is same between 2000 and 10000 SNP (**B**).

A The consensus tree with bootstrap with the block size 1000 SNPs is plotted. The bootstrap value is the number on each node of the tree and is the number of replicates that support the clade among the 100 iterations.

B The consensus tree with the window size of 2,000 SNPs with the bootstrap values. The only difference between the two phylogenies is the position of Malaysian Negritos. In the tree A, Malaysian Negritos is clustered together with Philippine Negritos. In the tree B, Malaysian Negritos is outside of Philippine Negritos with the higher bootstrap value (100) than the value in the tree A (70). Thus we chose the tree **B** for qpGraph modelling. The qpGraph results are similar enough between using the two different two topologies.

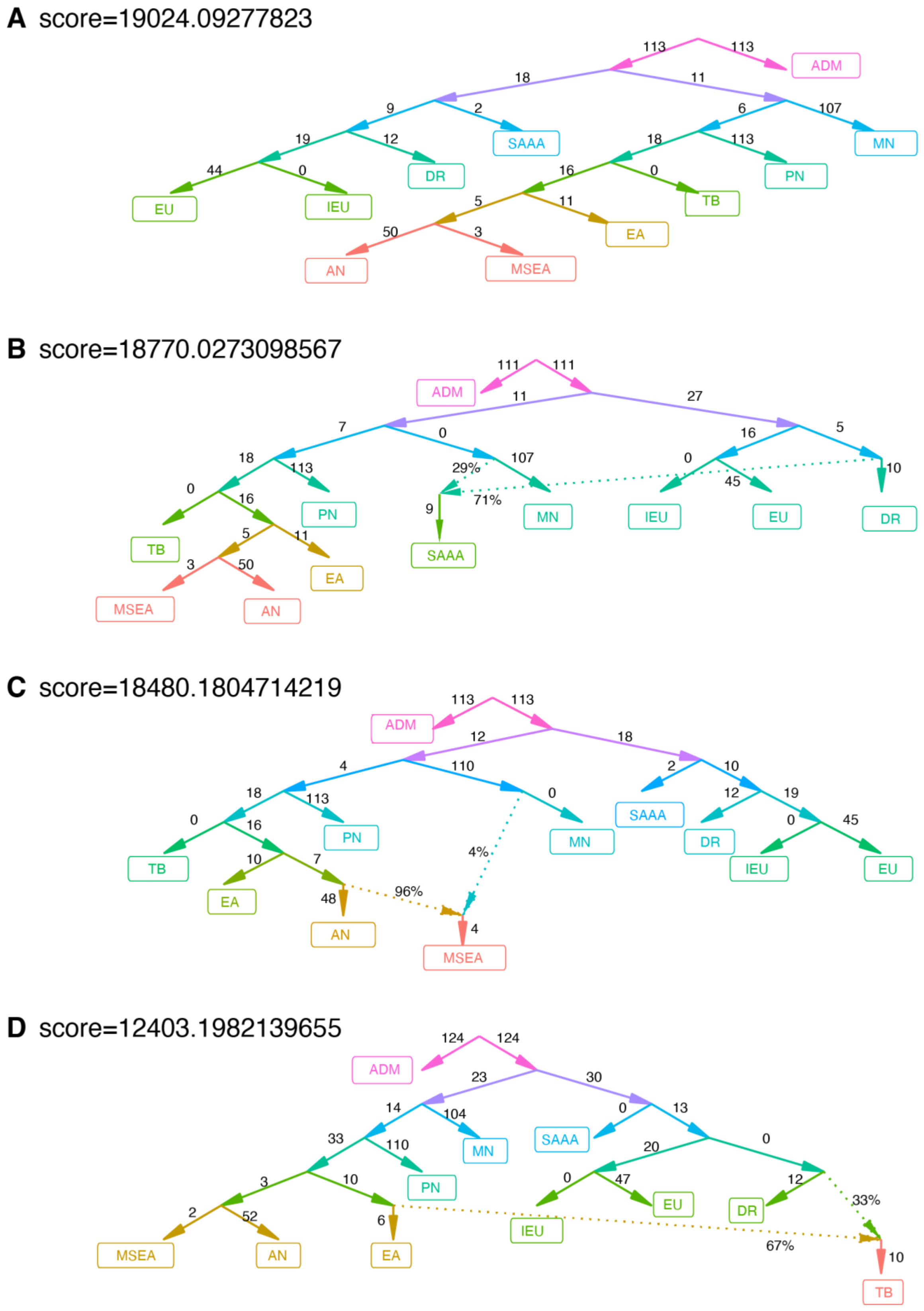
A score=19024.09277823
ADM
SAAA
MN
DR
PN
EU
IEU
TB
EA
AN
MSEA
B score=18770.0273098567
ADM
PN
MN
IEU
EU
DR
TB
SAAA
EA
MSEA
AN
29%
71%
C score=18480.1804714219
ADM
SAAA
PN
MN
DR
TB
IEU
EU
EA
AN
MSEA
4%
96%
D score=12403.1982139655
ADM
MN
SAAA
PN
EU
DR
IEU
MSEA
AN
EA
TB
33%
67%

E score=18720.31

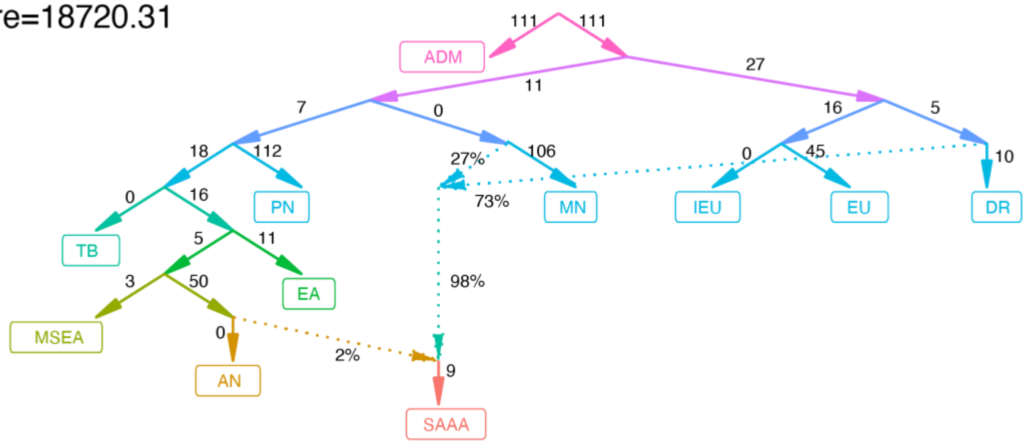

F score=18244.37

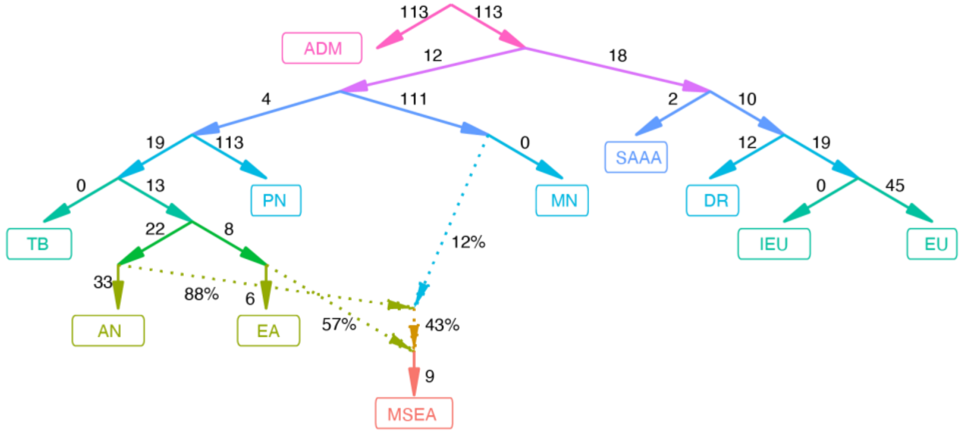

G score=12267.1

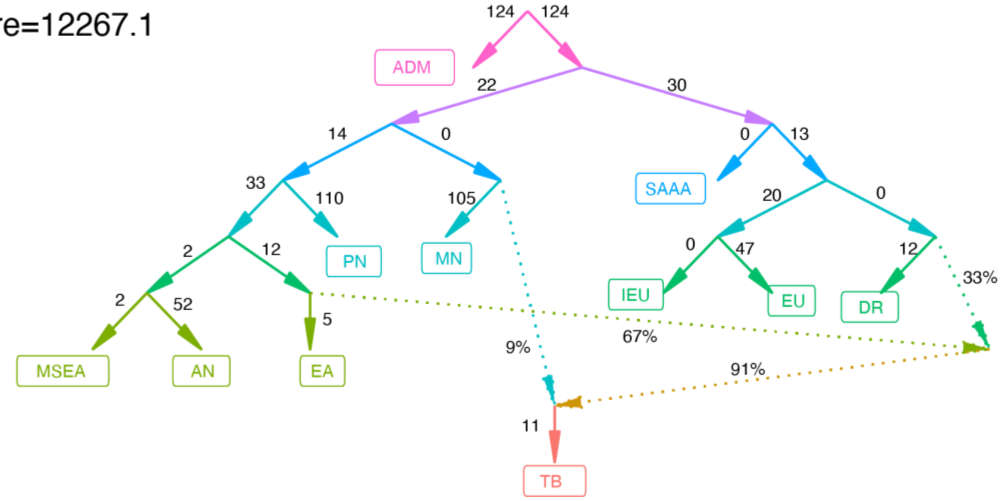

Supplementary Figure 10. Population genetic relationship modelling using qpGraph.
QpGraph modeling was performed using Admixtools 2[9]. We used the same set of data with Treemix, 630 individuals from 11 population groups and 919,892 SNPs. The base tree for running qpGraph was imported from the consensus tree of 100 iterations of Treemix with Andamanese as the outgroup (Supplementary Figure 9B). The name of the population groups in the graph are abbreviated as Andamanese (ADM), European (EU), Indo-European (IEU), Dravidian (DR), South Asian Austroasiatic (SAAA), Malaysian Negrito (MN), Philippine Negrito (PN), Austronesian (AN), Mainland Southeast Asian (MSEA), East Asian (EA), and Tibeto Burman (TB).

A The best-fit model without admixture event is plotted. Andamanese is the given outgroup for the consensus tree estimated by Treemix with no migration (Supplementary Figure 9B). The number on the branch is the drift lengths estimated by qpGraph.

To examine the admixture on **B** South Asian Austroasiatic, **C** Mainland Southeast Asian, and **D** Tibeto Burman population groups, we tested the 2-way admixture events for the three population groups as the target populations. We calculated the likelihood score of all pairs among 10 population groups for each of the three target population groups. There are 108 combinations of events that we tested, and the results are shown in Supplementary Table 4. The best-fit model to explain the South Asian Austroasiatic group (**B**) is admixture between Malaysian Negritos (71%) and Dravidian (29%) populations as shown in dotted lines. For Mainland Southeast Asians (**C**), the admixed edge coming from Malaysian Negritos and Austronesians have the lowest likelihood score with the admixture weight of 4% and 96%, respectively. For Tibeto Burman (**D**), the best-fit model is the admixture between Dravidians and East Asians with the admixture weight of 33% and 67%, respectively. We also performed the analysis with the other consensus tree (Supplementary Figure 9A) and found that the best-fit models for the target populations are same, as shown in Supplementary Table 5.

The best-fit model with 3-way admixture was calculated for the three target population groups, the South Asian Austroasiatic group **(E),** Mainland Southeast Asian (**F**), and Tibeto Burman **(G).** For the 3-way admixture, we added one more population group to the best-fitted 2-way admixture model and tested all combinations as shown in Supplementary Table 6.
E For the South Asian Austroasiatic group, the third best-fitted population group is Austronesian with 2% contribution to the admixture of Malaysian Negritos and Dravidian.
F Mainland Southeast Asians shows the best-fit with East Asian (57%) as third population groups to contribute in addition to Malaysian Negritos and Austronesians.
G Tibeto Burmans is best fitted with 9% of Malaysian Negrito contributions in addition to East Asians and Dravidians.

A. Admixture

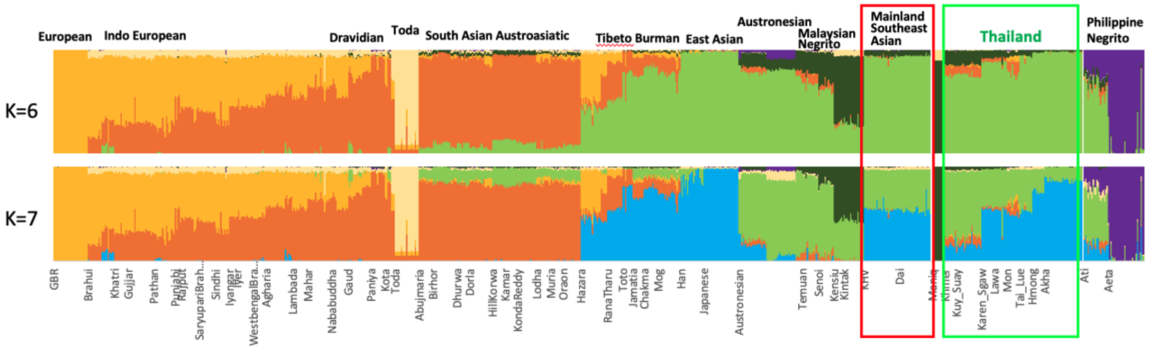

B. PCA

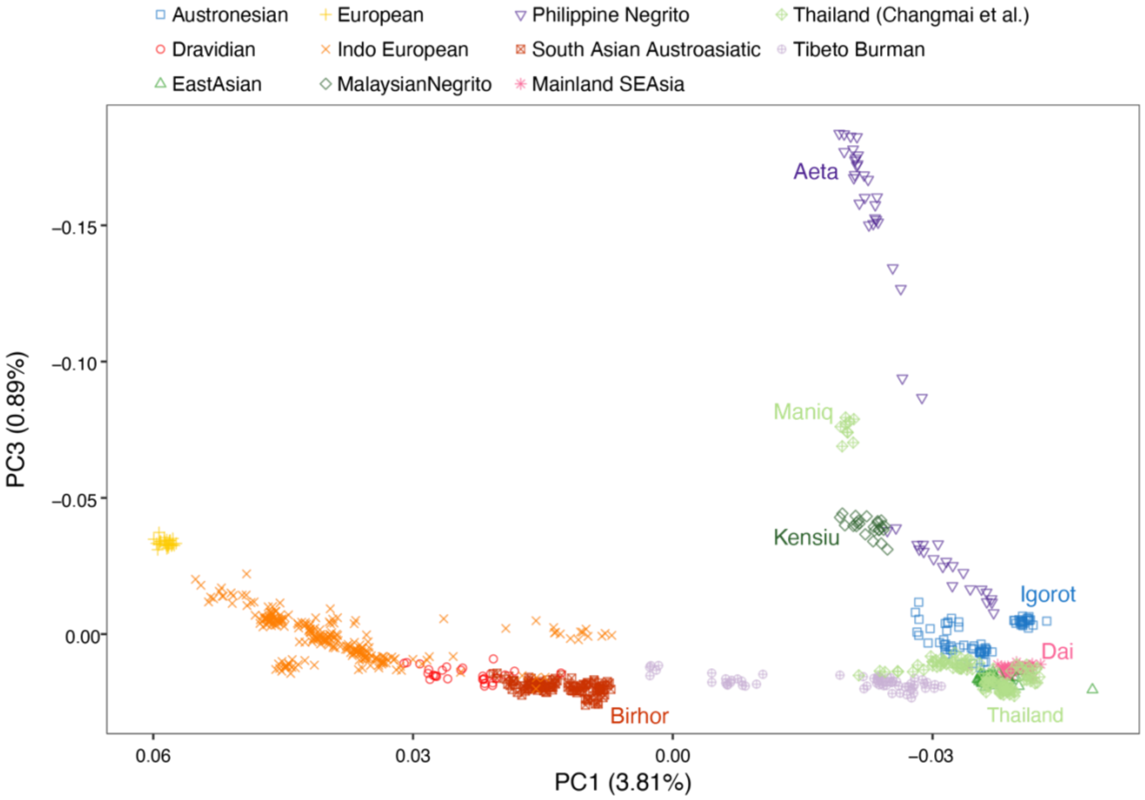

Supplementary Figure 11. Population structure analysis for Mainland Southeast Asian populations.

To fill the gap of Mainland Southeast Asian populations in our dataset, we retrieved the genotyping datasets of 120 individuals and 597,573 SNPs of mainly Thailand populations from the previous study[10]. We merge our datasets with the Thailand datasets based on the overlapping SNP positions. The merged datasets have 576,746 SNPs with 862 samples. The dataset was then filtered with PLINK1.9[2] with the following parameters: we included autosomes, individuals with missing genotype rate <0.1, SNPs with missing genotype <0.02, SNPs with MAF >0.01, and we pruned out SNPs with linkage r^2 >0.2 in a window size of 50kb with overlap 5 SNPs. The filtered datasets have 114,625 SNPs of 862 samples. Using this dataset we performed ADMIXTURE[1] (**A**) and PCA using PLINK (**B**).

Based on the results, we found that the KHV and Dai populations in our dataset (red line rectangular in **A**) show same major ancestral components with the diverse Thailand populations (green line rectangular). Only difference is some Northern Thailand populations have additional ancestry, South Asian component, which is a result of recent admixture[10]. The KHV and Dai have the minimum signal of the South Asian admixture. The PCA result also supports that our Mainland Southeast Asian populations, Dai and KHV, shown as pink stars in the plot (**B**), clustered together with most Thailand populations (light green diamond in the plot) except for the Negrito population, Maniq. Thus, we conclude that our Dai can be representative for Mainland Southeast Asians.

Supplementary Table 1. Global mean sea level (GMSL), its rate, and land size change history from 26 thousand years ago (KYA) till present.

A Global mean sea level (GMSL)

KYA	GMSL (m)
25	-130.59
24	-126.56
23	-124.13
22	-121.81
21	-120.17
20.5	-119.26
20	-118.34
19.5	-117.36
19	-116.40
18.5	-115.32
18	-113.52
17.5	-111.78
17	-109.95
16.5	-108.10
16	-105.73
15.5	-101.60
15	-97.80
14.5	-93.81
14	-70.90
13.5	-64.14
13	-59.47
12.5	-53.25
12	-48.89
11.5	-43.60
11	-32.68
10.5	-27.57
10	-22.36
9.5	-17.42
9	-13.56
8.5	-9.50
8	-6.47
7.5	-3.74
7	-2.35
6.5	-1.36
6	-0.97
5.5	-0.81
5	-0.62
4.5	-0.47
4	-0.25
3.5	-0.20
3	-0.16
2.5	-0.11
2	0
1.5	0
1	0
0.5	0
0	0

B GMSL rate

KYA	GMSL rate (mm/yr)
25.5	4.12
24.5	4.03
23.5	2.43
22.5	2.32
21.5	1.64
20.75	1.82
20.25	1.84
19.75	1.96
19.25	1.92
18.75	2.16
18.25	3.60
17.75	3.49
17.25	3.66
16.75	3.70
16.25	4.73
15.75	8.27
15.25	7.61
14.75	7.96
14.25	45.83
13.75	13.52
13.25	9.35
12.75	12.42
12.25	8.73
11.75	10.59
11.25	21.83
10.75	10.22
10.25	10.44
9.75	9.87
9.25	7.73
8.75	8.12
8.25	6.04
7.75	5.46
7.25	2.80
6.75	1.97
6.25	0.79
5.75	0.32
5.25	0.37
4.75	0.30
4.25	0.44
3.75	0.11
3.25	0.07
2.75	0.11
2.25	0.22
1.75	0
1.25	0
0.75	0
0.25	0

C Land size (Fig. 1C inset)

KYA	Land Size (x30.9136 km^2)
26	141,356
25	140,581
24	139,789
23	139,343
22	138,902
21	138,574
20	138,185
19	137,728
18	137,052
17	136,005
16	134,802
15	131,598
14	111,934
13	102,267
12	95,053
11	84,435
10	78,144
9	71,386
8	67,044
7	64,986
6	65,078
5	65,844
4	66,399
3	67,051
2	67,300
1	67,582
0	67,727

D Regional land size, effective population size, and population density. The effective population size (msmcN) was estimated by MSMC2[4,5] as shown in Supplementary Figure 6.

Kya	Island Southeast Asia (Fig. 4b blue inset)			Mainland Southeast Asia (Fig. 4b green inset)		
	Land Size (x30.9136 km^2)	msmcN	msmcN / Land size	Land Size (x30.9136 km^2)	msmcN	msmcN / Land size
26	109,184	7975	0.073	81,238	4437	0.055
25	108,560	7975	0.073	81,007	4437	0.055
24	107,866	7975	0.074	80,791	4437	0.055
23	107,493	7975	0.074	80,672	4437	0.055
22	107,114	14080	0.131	80,538	6068	0.075
21	106,833	14080	0.132	80,418	6068	0.075
20.5	106,662	14080	0.132	80,358	6068	0.076
20	106,491	14080	0.132	80,285	6068	0.076
19.5	106,276	14080	0.132	80,215	6068	0.076
19	106,105	14080	0.133	80,155	6068	0.076
18.5	105,884	14080	0.133	80,074	6068	0.076
18	105,502	14080	0.133	79,934	6068	0.076
17.5	105,116	14080	0.134	79,693	6068	0.076
17	105,047	14080	0.134	79,578	6068	0.076
16.5	104,862	14080	0.134	79,443	6068	0.076
16	104,502	14080	0.135	79,266	12635	0.159
15.5	103,480	25205	0.244	78,778	12635	0.160
15	101,823	25205	0.248	78,105	12635	0.162
14.5	100,611	25205	0.251	77,543	12635	0.163
14	87,064	25205	0.289	72,585	12635	0.174
13.5	82,240	25205	0.306	71,313	12635	0.177
13	78,774	25205	0.320	70,293	12635	0.180
12.5	74,431	25205	0.339	68,870	12635	0.183
12	72,084	25205	0.350	67,824	12635	0.186
11.5	69,433	25205	0.363	66,248	12635	0.191
11	63,490	25205	0.397	63,316	12635	0.200
10.5	61,255	25205	0.411	61,763	29724	0.481
10	58,955	32028	0.543	60,202	29724	0.494
9.5	56,718	32028	0.565	58,669	29724	0.507
9	54,894	32028	0.583	57,259	29724	0.519
8.5	52,693	32028	0.608	55,411	29724	0.536
8	51,831	32028	0.618	54,708	29724	0.543
7.5	51,096	32028	0.627	53,492	29724	0.556
7	50,838	32028	0.630	53,099	29724	0.560
6.5	50,665	32028	0.632	52,966	29724	0.561
6	50,796	1466	0.029	53,149	29350	0.552
5.5	50,993	1466	0.029	53,433	29350	0.549
5	51,139	1466	0.029	53,670	29350	0.547
4.5	51,251	1466	0.029	53,883	29350	0.545
4	51,346	1466	0.029	54,087	29350	0.543

3.5	51,442	1466	0.028	54,273	29350	0.541
3	51,553	1466	0.028	54,401	29350	0.540
2.5	51,639	1466	0.028	54,450	29350	0.539
2	51,701	1466	0.028	54,487	29350	0.539
1.5	51,767	1466	0.028	54,550	29350	0.538
1	51,805	1466	0.028	54,604	29350	0.537
0.5	51,902	1466	0.028	54,671	29350	0.537
0	51,902	1466	0.028	54,671	29350	0.537

Supplementary Table 2. The 763 whole genome datasets are a portion of the GenomeAsia 100K pilot dataset[3]. The table shows the number of samples for each population used in this study.

Group	Population	No. of Samples
European	GBR (British in England and Scotland)	27
Indo European	Brahui	11
	Pathan	17
	Brusho	10
	Hazara	16
	Sindhi	11
	Gujjar	20
	Khatri	11
	Punjabi	5
	Rajput	14
	Saryupari Brahmin	13
	West Bengal Brahmin	10
	Iyangar	6
	Iyer	13
	Saurashtra Brahmin	9
	Agharia	15
	Lambada	12
	Chamar	6
	Nababuddha	4
	Mahar	19
	Kota	8
	Toda	19
Dravidian	Irula	4
	Chanchu	7
	Paniya	11
	Bagdi	3
	Gaud	4
South Asian Austroasiatic	Halba	6
	Lodha	11
	Abujmaria	11
	Bison Horn Maria	5
	Muria	10
	Oraon	14
	Dorla	12
	Dhurwa	10
	Konda Reddy	15
	Kamar	10
	Hill Korwa	10
	Birhor	14
Tibeto Burman	Manipuri	5
	Rana Tharu	12
	Toto	8
	Jamatia	9
	Mog	18
	Chakma	11
Malaysian Negrito	Kensiu	8
	Kintak	16
Austronesian	Senoi	12
	Temuan	13
	Malay Singaporean	20
	Mentawai/Nias	23

	Igorot	2
	Nicobarese	5
Philippine Negrito	Ati	19
	Aeta	29
Mainland Southeast Asian	Dai	25
	KHV (Kinh in Ho Chi Minh)	28
East Asian	Han Chinese	18
	Japanese	28
Andamanese	Jarwa	10
	Onge	11
**TOTAL**	**61**	**763**

Supplementary Table 3. Population split time (years ago) estimated based on *M(t),* that is the cumulative migration probability that denotes the probability for lineages to have merged by the time *t,* at quantile for a pair of populations, estimated using MSMC-IM[6]. For each pair, we performed four to five pairs of different combinations of individual genomes to see the robustness of estimates. *M(t)* is the cumulative migration probability that denotes the probability for lineages to have merged by the time *t*. Supplementary Figure 3 shows the plots from these tables and includes one estimate for each pair of populations.

A The time *t* between **a pair of populations within Southeast Asians** for each MSMC run is shown in the table for three *M(t)* quantile = 0.25, 0.5, and 0.75. The time between Kensiu and Dai supposes to be similar to the time of two other pairs, Kensiu-Aeta and Kensiu-Igorot, as shown at *M(t)* = 0.75, but is younger at *M(t)* = 0.5 and 0.25. Kensiu-Dai results are similar with Kensiu-KHV (purple shaded).

M(t) quantile	0.25	0.5	0.75
**Kensiu-Han**	10,223	14,292	22,171
	10,290	14,472	22,314
	11,214	15,850	23,098
	10,886	15,836	23,048
**Kensiu-Aeta**	10,673	15,361	21,291
	8,763	14,319	21,127
	9,687	14,290	20,084
	9,240	13,693	20,969
	7,909	12,712	20,288
**Kensiu-Igorot**	9,590	14,501	22,300
	9,778	14,460	21,919
	9,863	14,352	22,052
	9,830	13,860	22,136
**Kensiu-Dai**	6,970	12,013	21,888
	7,009	10,974	20,748
	6,657	10,746	21,545
	6,843	10,534	21,045
	6,639	9,299	20,973
**Kensiu-KHV**	6,711	10,944	21,155
	7,158	11,758	21,938
	6,536	8,514	18,764
	6,344	7,974	23,026
**Igorot-Dai**	7,093	10,615	14,565
	7,234	10,394	14,765
	6,956	10,228	14,865
	6,926	9,922	14,328
	6,734	9,682	14,113

B M(t) estimates between **a pair of populations within South Asians** populations

M(t) quantile	0.25	0.5	0.75
**Birhor-Gujjar**	7,316	12,894	19,208
	7,491	11,791	19,619
	7,520	11,366	20,810

	6,956	10,480	19,346
	7,008	10,186	21,341
**Paniya-Gujjar**	7,561	11,738	21,120
	7,341	11,360	21,073
	7,381	11,010	18,878
	7,143	10,253	19,113
	7,111	9,405	16,861
**Birhor-Paniya**	6,632	7,892	13,566
	6,543	7,816	13,817
	6,514	7,729	13,333
	6,485	7,680	11,932
	6,378	7,573	11,955

C The *t* between a pair of populations **between Birhor and Southeast Asian** populations for each MSMC run is shown in the table for three *M(t)* quantile = 0.25, 0.5, and 0.75. The time between Birhor and Kensiu supposes to be older as similar as the pairs between Birhor-Igorot, Birhor-Aeta, and Birhor-Dai. However, the time is younger (purple shaded) as similar to the split within South Asian populations such as Birhor-Gujjar.

M(t) quantile	0.25	0.5	0.75
**Birhor-Igorot**	13,4 49	17,813	25,317
	13,305	17,773	25,891
	13,143	17,591	25,521
	12,349	17,345	25,621
	13,110	17,159	25,590
**Birhor-Aeta**	11,246	16,199	23,219
	11,067	16,176	23,377
	11,340	15,956	23,173
	10,794	15,755	24,107
	11,473	15,455	21,461
**Birhor-Dai**	10,460	17,399	24,402
	10,613	16,250	26,235
	9,450	15,893	24,006
	8,919	15,455	23,547
	10,333	15,417	23,493
**Birhor-KHV**	10,749	16,007	23,086
	7,536	14,268	24,154
	10,333	16,671	23,838
**Birhor-Kensiu**	8,222	13,112	20,227
	7,450	12,267	21,363
	7,699	11,991	19,881
	7,538	11,581	19,495
	7,400	11,093	20,519

D M(t) estimates between **Jamatia (Tibeto Burman) and other Asians.** Migration after split between Jamatia and Dai/Han has been prolonged after their splits and the time at M(t)=0.25 is younger compared to the Jamatia-Igorot pair.

M(t) quantile	0.25	0.5	0.75
	11,188	17,529	25,182
**Jamatia-Gujjar**	12,173	17,174	25,635
	12,023	17,263	25,289
	9,225	14,089	22,251
**Jamatia-Paniya**	9,047	14,120	22,660
	8,323	13,868	21,961
	7,960	13,471	19,357
**Jamatia-Birhor**	7,625	13,099	22,393
	7,502	12,105	19,938
	9,464	13,073	19,233
**Jamatia-Aeta**	9,296	13,887	21,415
	8,566	12,815	20,492
	6,988	11,507	19,454
**Jamatia-Kensiu**	6,788	9,314	19,174
	6,643	8,479	17,679
	9,813	13,217	17,566
**Jamatia-Igorot**	9,649	12,976	18,027
	9,264	12,378	17,390
	5,179	6,884	10,662
**Jamatia-Dai**	5,581	7,876	11,957
	5,406	9,154	13,785
	4,816	7,506	11,413
**Jamatia-Han**	2,711	6,690	11,075
	2,510	6,975	11,264

Supplementary Table 4. QpGraph 2-way admixture likelihood scores with the consensus tree of Supplementary Figure 9B. The name of the population groups in the graph are abbreviated: South Asian Austroasiatic (SAAA) and Mainland Southeast Asian (MSEA). The best fitted model, having the lowest likelihood score (score), is shaded by purple, for each admixed population, is plotted in Supplementary Figure 10.

pop1	pop2	Admixed pop	score	weight.pop1	weight.pop2
Dravidian	Malaysian Negrito	SAAA	18770.0	0.7082	0.2918
Dravidian	Philippine Negrito	SAAA	18821.6	0.7486	0.2514
Dravidian	TibetoBurman	SAAA	18992.3	0.8334	0.1666
Dravidian	EastAsian	SAAA	19509.7	0.8840	0.1160
Dravidian	Austronesian	SAAA	19527.8	0.8927	0.1073
Dravidian	MSEA	SAAA	19527.9	0.8925	0.1075
Dravidian	European	SAAA	20326.1	1.0000	0.0000
Dravidian	IndoEuropean	SAAA	20326.1	1.0000	0.0000
European	Malaysian Negrito	SAAA	22139.8	0.3244	0.6756
IndoEuropean	Malaysian Negrito	SAAA	22139.8	0.3244	0.6756
Austronesian	Malaysian Negrito	SAAA	22641.5	0.0129	0.9871
MSEA	Malaysian Negrito	SAAA	22667.4	0.0046	0.9954
Philippine Negrito	Malaysian Negrito	SAAA	22668.6	0.0000	1.0000
EastAsian	Malaysian Negrito	SAAA	22668.7	0.0000	1.0000
TibetoBurman	Malaysian Negrito	SAAA	22668.7	0.0000	1.0000
European	Philippine Negrito	SAAA	22839.4	0.4825	0.5175
IndoEuropean	Philippine Negrito	SAAA	22839.4	0.4825	0.5175
European	TibetoBurman	SAAA	24240.8	0.7288	0.2712
IndoEuropean	TibetoBurman	SAAA	24240.8	0.7288	0.2712
Austronesian	Philippine Negrito	SAAA	24768.3	0.0117	0.9883
MSEA	Philippine Negrito	SAAA	24789.4	0.0025	0.9975
EastAsian	Philippine Negrito	SAAA	24789.7	0.0000	1.0000
TibetoBurman	Philippine Negrito	SAAA	24789.8	0.0000	1.0000
IndoEuropean	EastAsian	SAAA	25262.2	0.8361	0.1639
European	EastAsian	SAAA	25262.2	0.8361	0.1639
IndoEuropean	Austronesian	SAAA	25360.3	0.8488	0.1512
European	Austronesian	SAAA	25360.3	0.8488	0.1512
IndoEuropean	MSEA	SAAA	25360.3	0.8488	0.1512
European	MSEA	SAAA	25360.3	0.8488	0.1512
IndoEuropean	European	SAAA	26988.6	0.6544	0.3456
TibetoBurman	Austronesian	SAAA	31339.4	1.0000	0.0000
TibetoBurman	EastAsian	SAAA	31339.5	1.0000	0.0000
TibetoBurman	MSEA	SAAA	31339.5	1.0000	0.0000
Austronesian	EastAsian	SAAA	32779.0	0.0091	0.9909
MSEA	EastAsian	SAAA	32779.7	0.0189	0.9811
MSEA	Austronesian	SAAA	33603.4	0.4460	0.5540
Austronesian	Malaysian Negrito	MSEA	18480.2	0.9560	0.0440
Austronesian	EastAsian	MSEA	18826.9	0.3837	0.6163
Austronesian	Philippine Negrito	MSEA	18842.4	0.9800	0.0200
European	Austronesian	MSEA	18934.1	0.0000	1.0000
Dravidian	Austronesian	MSEA	18934.1	0.0000	1.0000
TibetoBurman	Austronesian	MSEA	18934.1	0.0000	1.0000

SAAA	Austronesian	MSEA	18934.1	0.0000	1.0000
IndoEuropean	Austronesian	MSEA	18934.1	0.0000	1.0000
EastAsian	Malaysian Negrito	MSEA	19065.7	0.9672	0.0328
EastAsian	Philippine Negrito	MSEA	19316.2	0.9915	0.0085
European	EastAsian	MSEA	19329.8	0.0000	1.0000
Dravidian	EastAsian	MSEA	19329.8	0.0000	1.0000
TibetoBurman	EastAsian	MSEA	19329.9	0.0000	1.0000
SAAA	EastAsian	MSEA	19329.9	0.0000	1.0000
IndoEuropean	EastAsian	MSEA	19329.9	0.0000	1.0000
TibetoBurman	Malaysian Negrito	MSEA	23964.1	0.9781	0.0219
TibetoBurman	Philippine Negrito	MSEA	24065.5	1.0000	0.0000
European	TibetoBurman	MSEA	24065.6	0.0000	1.0000
Dravidian	TibetoBurman	MSEA	24065.6	0.0000	1.0000
SAAA	TibetoBurman	MSEA	24065.6	0.0000	1.0000
IndoEuropean	TibetoBurman	MSEA	24065.6	0.0000	1.0000
Philippine Negrito	Malaysian Negrito	MSEA	25701.2	0.9782	0.0218
European	Philippine Negrito	MSEA	25791.7	0.0000	1.0000
Dravidian	Philippine Negrito	MSEA	25791.8	0.0000	1.0000
SAAA	Philippine Negrito	MSEA	25791.8	0.0000	1.0000
IndoEuropean	Philippine Negrito	MSEA	25791.8	0.0000	1.0000
European	Malaysian Negrito	MSEA	26705.6	0.0000	1.0000
Dravidian	Malaysian Negrito	MSEA	26705.6	0.0000	1.0000
SAAA	Malaysian Negrito	MSEA	26705.7	0.0000	1.0000
IndoEuropean	Malaysian Negrito	MSEA	26705.7	0.0000	1.0000
SAAA	European	MSEA	33690.4	1.0000	0.0000
SAAA	Dravidian	MSEA	33690.4	1.0000	0.0000
SAAA	IndoEuropean	MSEA	33690.5	1.0000	0.0000
Dravidian	European	MSEA	34119.5	1.0000	0.0000
Dravidian	IndoEuropean	MSEA	34119.6	1.0000	0.0000
IndoEuropean	European	MSEA	39020.5	0.3427	0.6573

Dravidian	EastAsian	TibetoBurman	12403.2	0.3324	0.6676
SAAA	EastAsian	TibetoBurman	12824.9	0.3524	0.6476
European	EastAsian	TibetoBurman	13092.3	0.2919	0.7081
IndoEuropean	EastAsian	TibetoBurman	13101.1	0.2922	0.7078
Dravidian	Austronesian	TibetoBurman	13932.3	0.3155	0.6845
Dravidian	MSEA	TibetoBurman	13932.4	0.3155	0.6845
SAAA	Austronesian	TibetoBurman	14312.3	0.3330	0.6670
SAAA	MSEA	TibetoBurman	14312.4	0.3330	0.6670
European	Austronesian	TibetoBurman	14583.7	0.2787	0.7213
European	MSEA	TibetoBurman	14583.7	0.2787	0.7213
IndoEuropean	Austronesian	TibetoBurman	14591.4	0.2790	0.7210
IndoEuropean	MSEA	TibetoBurman	14591.4	0.2790	0.7210
EastAsian	Malaysian Negrito	TibetoBurman	15611.6	0.4967	0.5033
Dravidian	Philippine Negrito	TibetoBurman	16237.0	0.2620	0.7380
European	Philippine Negrito	TibetoBurman	16249.1	0.2209	0.7791
IndoEuropean	Philippine Negrito	TibetoBurman	16258.2	0.2211	0.7789
SAAA	Philippine Negrito	TibetoBurman	16820.0	0.2796	0.7204
Austronesian	Malaysian Negrito	TibetoBurman	17071.9	0.5331	0.4669
MSEA	Malaysian Negrito	TibetoBurman	17071.9	0.5331	0.4669

EastAsian	Philippine Negrito	TibetoBurman	17669.1	0.4596	0.5404
European	Malaysian Negrito	TibetoBurman	18520.1	0.1802	0.8198
IndoEuropean	Malaysian Negrito	TibetoBurman	18523.3	0.1801	0.8199
Dravidian	Malaysian Negrito	TibetoBurman	18691.5	0.2198	0.7802
Philippine Negrito	Malaysian Negrito	TibetoBurman	18885.1	0.5632	0.4368
Austronesian	Philippine Negrito	TibetoBurman	18941.1	0.5051	0.4949
MSEA	Philippine Negrito	TibetoBurman	18941.1	0.5051	0.4949
SAAA	Malaysian Negrito	TibetoBurman	19228.1	0.2284	0.7716
Austronesian	EastAsian	TibetoBurman	22901.6	0.0000	1.0000
MSEA	EastAsian	TibetoBurman	22901.7	0.0000	1.0000
MSEA	Austronesian	TibetoBurman	24065.6	0.5518	0.4482
SAAA	European	TibetoBurman	30138.1	0.9586	0.0414
SAAA	IndoEuropean	TibetoBurman	30138.1	0.9586	0.0414
SAAA	Dravidian	TibetoBurman	30175.8	1.0000	0.0000
Dravidian	European	TibetoBurman	30740.1	0.9687	0.0313
Dravidian	IndoEuropean	TibetoBurman	30740.2	0.9687	0.0313
IndoEuropean	European	TibetoBurman	36039.4	0.4375	0.5625

Supplementary Table 5. QpGraph 2-way likelihood scores with the consensus tree of Supplementary Figure 9A. The name of the population groups in the graph are abbreviated: South Asian Austroasiatic (SAAA) and Mainland Southeast Asian (MSEA). The best fitted model, having the lowest likelihood score (score), is shaded by purple.

pop1	pop2	Admixed pop	score	weight.pop1	weight.pop2
Dravidian	MalaysianNegrito	SAAA	18947.1	0.7351	0.2649
Dravidian	PhilippineNegrito	SAAA	18948.7	0.7334	0.2666
Dravidian	TibetoBurman	SAAA	19139.2	0.8313	0.1687
Dravidian	EastAsian	SAAA	19669.7	0.8834	0.1166
Dravidian	Austronesian	SAAA	19689.8	0.8921	0.1079
Dravidian	MSEA	SAAA	19689.8	0.8920	0.1080
Dravidian	European	SAAA	20478.2	1.0000	0.0000
Dravidian	IndoEuropean	SAAA	20478.2	1.0000	0.0000
European	MalaysianNegrito	SAAA	22839.7	0.4811	0.5189
European	PhilippineNegrito	SAAA	22839.7	0.4811	0.5189
IndoEuropean	MalaysianNegrito	SAAA	22839.7	0.4811	0.5189
IndoEuropean	PhilippineNegrito	SAAA	22839.7	0.4811	0.5189
European	TibetoBurman	SAAA	24343.9	0.7141	0.2859
IndoEuropean	TibetoBurman	SAAA	24343.9	0.7141	0.2859
Austronesian	MalaysianNegrito	SAAA	24768.3	0.0117	0.9883
Austronesian	PhilippineNegrito	SAAA	24768.3	0.0117	0.9883
MSEA	MalaysianNegrito	SAAA	24789.4	0.0025	0.9975
MSEA	PhilippineNegrito	SAAA	24789.4	0.0025	0.9975
PhilippineNegrito	MalaysianNegrito	SAAA	24789.7	0.4958	0.5042
EastAsian	MalaysianNegrito	SAAA	24789.8	0.0000	1.0000
EastAsian	PhilippineNegrito	SAAA	24789.8	0.0000	1.0000
TibetoBurman	MalaysianNegrito	SAAA	24789.8	0.0000	1.0000
TibetoBurman	PhilippineNegrito	SAAA	24789.8	0.0000	1.0000
European	EastAsian	SAAA	25452.6	0.8286	0.1714
IndoEuropean	EastAsian	SAAA	25452.6	0.8286	0.1714

IndoEuropean	Austronesian	SAAA	25558.8	0.8459	0.1541
European	Austronesian	SAAA	25558.8	0.8459	0.1541
IndoEuropean	MSEA	SAAA	25558.8	0.8459	0.1541
European	MSEA	SAAA	25558.8	0.8459	0.1541
European	IndoEuropean	SAAA	27193.2	0.3456	0.6544
Austronesian	TibetoBurman	SAAA	31326.9	0.0000	1.0000
EastAsian	TibetoBurman	SAAA	31326.9	0.0000	1.0000
MSEA	TibetoBurman	SAAA	31326.9	0.0000	1.0000
Austronesian	EastAsian	SAAA	32730.2	0.0089	0.9911
MSEA	EastAsian	SAAA	32730.7	0.0192	0.9808
Austronesian	MSEA	SAAA	33534.3	0.5540	0.4460
Austronesian	MalaysianNegrito	MSEA	18560.2	0.9532	0.0468
Austronesian	EastAsian	MSEA	18961.9	0.3848	0.6152
Austronesian	PhilippineNegrito	MSEA	18976.5	0.9799	0.0201
European	Austronesian	MSEA	19073.0	0.0000	1.0000
Dravidian	Austronesian	MSEA	19073.0	0.0000	1.0000
Austronesian	TibetoBurman	MSEA	19073.1	1.0000	0.0000
SAAA	Austronesian	MSEA	19073.1	0.0000	1.0000
IndoEuropean	Austronesian	MSEA	19073.1	0.0000	1.0000
EastAsian	MalaysianNegrito	MSEA	19146.1	0.9645	0.0355
EastAsian	PhilippineNegrito	MSEA	19437.3	0.9914	0.0086
European	EastAsian	MSEA	19455.0	0.0000	1.0000
Dravidian	EastAsian	MSEA	19455.0	0.0000	1.0000
EastAsian	TibetoBurman	MSEA	19455.0	1.0000	0.0000
SAAA	EastAsian	MSEA	19455.0	0.0000	1.0000
IndoEuropean	EastAsian	MSEA	19455.1	0.0000	1.0000
TibetoBurman	MalaysianNegrito	MSEA	23997.7	0.9764	0.0236
TibetoBurman	PhilippineNegrito	MSEA	24115.6	1.0000	0.0000
European	TibetoBurman	MSEA	24115.6	0.0000	1.0000
Dravidian	TibetoBurman	MSEA	24115.7	0.0000	1.0000
SAAA	TibetoBurman	MSEA	24115.7	0.0000	1.0000
IndoEuropean	TibetoBurman	MSEA	24115.7	0.0000	1.0000
PhilippineNegrito	MalaysianNegrito	MSEA	26705.5	0.0000	1.0000
European	MalaysianNegrito	MSEA	26705.5	0.0000	1.0000
Dravidian	MalaysianNegrito	MSEA	26705.6	0.0000	1.0000
SAAA	MalaysianNegrito	MSEA	26705.6	0.0000	1.0000
IndoEuropean	MalaysianNegrito	MSEA	26705.6	0.0000	1.0000
European	PhilippineNegrito	MSEA	26866.9	0.0000	1.0000
Dravidian	PhilippineNegrito	MSEA	26867.0	0.0000	1.0000
SAAA	PhilippineNegrito	MSEA	26867.0	0.0000	1.0000
IndoEuropean	PhilippineNegrito	MSEA	26867.0	0.0000	1.0000
SAAA	European	MSEA	33641.3	1.0000	0.0000
SAAA	Dravidian	MSEA	33641.3	1.0000	0.0000
SAAA	IndoEuropean	MSEA	33641.4	1.0000	0.0000
Dravidian	European	MSEA	34073.1	1.0000	0.0000
Dravidian	IndoEuropean	MSEA	34073.2	1.0000	0.0000
European	IndoEuropean	MSEA	38973.5	0.6569	0.3431
Dravidian	EastAsian	TibetoBurman	12941.9	0.3293	0.6707

SAAA	EastAsian	TibetoBurman	13374.7	0.3496	0.6504
European	EastAsian	TibetoBurman	13592.0	0.2879	0.7121
IndoEuropean	EastAsian	TibetoBurman	13600.3	0.2882	0.7118
Dravidian	Austronesian	TibetoBurman	14451.1	0.3118	0.6882
Dravidian	MSEA	TibetoBurman	14451.2	0.3118	0.6882
SAAA	Austronesian	TibetoBurman	14844.3	0.3294	0.6706
SAAA	MSEA	TibetoBurman	14844.3	0.3294	0.6706
European	Austronesian	TibetoBurman	15059.7	0.2743	0.7257
European	MSEA	TibetoBurman	15059.7	0.2743	0.7257
IndoEuropean	Austronesian	TibetoBurman	15066.8	0.2746	0.7254
IndoEuropean	MSEA	TibetoBurman	15066.9	0.2746	0.7254
EastAsian	MalaysianNegrito	TibetoBurman	17845.5	0.5006	0.4994
EastAsian	PhilippineNegrito	TibetoBurman	17845.5	0.5006	0.4994
European	MalaysianNegrito	TibetoBurman	18811.8	0.2065	0.7935
European	PhilippineNegrito	TibetoBurman	18811.8	0.2065	0.7935
IndoEuropean	MalaysianNegrito	TibetoBurman	18819.1	0.2066	0.7934
IndoEuropean	PhilippineNegrito	TibetoBurman	18819.1	0.2066	0.7934
Dravidian	MalaysianNegrito	TibetoBurman	18874.1	0.2462	0.7538
Dravidian	PhilippineNegrito	TibetoBurman	18874.1	0.2462	0.7538
Austronesian	MalaysianNegrito	TibetoBurman	19108.4	0.5424	0.4576
Austronesian	PhilippineNegrito	TibetoBurman	19108.4	0.5424	0.4576
MSEA	MalaysianNegrito	TibetoBurman	19108.4	0.5424	0.4576
MSEA	PhilippineNegrito	TibetoBurman	19108.4	0.5424	0.4576
SAAA	MalaysianNegrito	TibetoBurman	19433.0	0.2622	0.7378
SAAA	PhilippineNegrito	TibetoBurman	19433.0	0.2622	0.7378
PhilippineNegrito	MalaysianNegrito	TibetoBurman	22516.3	0.4889	0.5111
Austronesian	EastAsian	TibetoBurman	22968.0	0.0000	1.0000
MSEA	EastAsian	TibetoBurman	22968.0	0.0000	1.0000
Austronesian	MSEA	TibetoBurman	24115.6	0.4482	0.5518
SAAA	European	TibetoBurman	30212.4	0.9562	0.0438
SAAA	IndoEuropean	TibetoBurman	30212.5	0.9562	0.0438
SAAA	Dravidian	TibetoBurman	30254.9	1.0000	0.0000
Dravidian	European	TibetoBurman	30809.8	0.9659	0.0341
Dravidian	IndoEuropean	TibetoBurman	30809.9	0.9659	0.0341
European	IndoEuropean	TibetoBurman	36086.9	0.5625	0.4375

Supplementary Table 6. QpGraph 3-way admixture likelihood scores with the consensus tree (Supplementary Figure 9B). The name of the population groups in the graph are abbreviated: South Asian Austroasiatic (SAAA) and Mainland Southeast Asian (MSEA). The best fitted model, having the lowest likelihood score (score), is shaded by purple, for each admixed population, is plotted in Supplementary Figure 10.

pop1	pop2	pop3	Admixed pop	score	Weight pop1	Weight pop2	weight.2 pop3	weight.2 (pop1.pop2)
Dravidian	Malaysian Negrito	Austronesian	SAAA	18720.31	0.7265	0.2735	0.0180	0.9820
Dravidian	Malaysian Negrito	Tibeto Burman	SAAA	18740.83	0.7768	0.2232	0.0559	0.9441
Dravidian	Malaysian Negrito	MSEA	SAAA	18748.39	0.7305	0.2695	0.0212	0.9788
Dravidian	Malaysian Negrito	East Asian	SAAA	18765.56	0.7204	0.2796	0.0116	0.9884
Dravidian	Malaysian Negrito	Philippine Negrito	SAAA	18769.98	0.7082	0.2918	0.0000	1.0000
Dravidian	Malaysian Negrito	European	SAAA	18770.01	0.7082	0.2918	0.0000	1.0000

Dravidian	Malaysian Negrito	Indo-European	SAAA	18770.08	0.7082	0.2918	0.0000	1.0000
Austronesian	Malaysian Negrito	East Asian	MSEA	18244.37	0.8815	0.1185	0.5695	0.4305
Austronesian	Malaysian Negrito	Philippine Negrito	MSEA	18429.41	0.9572	0.0428	0.0149	0.9851
Austronesian	Malaysian Negrito	European	MSEA	18480.16	0.9560	0.0440	0.0000	1.0000
Austronesian	Malaysian Negrito	Dravidian	MSEA	18480.18	0.9560	0.0440	0.0000	1.0000
Austronesian	Malaysian Negrito	Tibeto Burman	MSEA	18480.21	0.9560	0.0440	0.0000	1.0000
Austronesian	Malaysian Negrito	SAAA	MSEA	18480.22	0.9560	0.0440	0.0000	1.0000
Austronesian	Malaysian Negrito	Indo-European	MSEA	18480.24	0.9560	0.0440	0.0000	1.0000
Dravidian	East Asian	Malaysian Negrito	Tibeto Burman	12267.10	0.3263	0.6737	0.0947	0.9053
Dravidian	East Asian	European	Tibeto Burman	12267.30	0.2622	0.7378	0.0868	0.9132
Dravidian	East Asian	Indo-European	Tibeto Burman	12267.38	0.2623	0.7377	0.0867	0.9133
Dravidian	East Asian	Philippine Negrito	Tibeto Burman	12311.86	0.3391	0.6609	0.0866	0.9134
Dravidian	East Asian	SAAA	Tibeto Burman	12314.14	0.2632	0.7368	0.1054	0.8946
Dravidian	East Asian	MSEA	Tibeto Burman	12401.87	0.4245	0.5755	0.2004	0.7996
Dravidian	East Asian	Austronesian	Tibeto Burman	12403.17	0.3324	0.6676	0.0000	1.0000

References

1. Alexander, D. H., Novembre, J. & Lange, K. Fast model-based estimation of ancestry in unrelated individuals. *Genome Res* **19**, 1655–1664 (2009).
2. Chang, C. C. *et al.* Second-generation PLINK: rising to the challenge of larger and richer datasets. *Gigascience* **4**, 7 (2015).
3. GenomeAsia100K Consortium. The GenomeAsia 100K Project enables genetic discoveries across Asia. *Nature* **576**, 106–111 (2019).
4. Schiffels, S. & Durbin, R. Inferring human population size and separation history from multiple genome sequences. *Nat Genet* **46**, 919–925 (2014).
5. Schiffels, S. & Wang, K. MSMC and MSMC2: The Multiple Sequentially Markovian Coalescent. in *Statistical Population Genomics* (ed. Dutheil, J. Y.) 147–166 (Springer US, 2020). doi:10.1007/978-1-0716-0199-0_7.
6. Wang, K., Mathieson, I., O'Connell, J. & Schiffels, S. Tracking human population structure through time from whole genome sequences. *PLoS Genet* **16**, e1008552 (2020).
7. Pickrell, J. K. & Pritchard, J. K. Inference of Population Splits and Mixtures from Genome-Wide Allele Frequency Data. *PLoS Genet* **8**, e1002967 (2012).
8. FELSENSTEIN, J. PHYLIP-Phylogeny interference package (version 3.2). *Cladistics* **5**, 164–166 (1989).
9. Patterson, N. *et al.* Ancient Admixture in Human History. *Genetics* **192**, 1065–1093 (2012).
10. Changmai, P. *et al.* Indian genetic heritage in Southeast Asian populations. *PLoS Genet* **18**, e1010036 (2022).
