# Supplementary material for: Prehistoric human migration between Sundaland and South Asia was driven by sea-level rise
Source: Commun Biol. 2023 Feb 4;6:150. doi: 10.1038/s42003-023-04510-0 (PMC9899273; doi:10.1038/s42003-023-04510-0)
Supplement: Supplementary file 2 — Description of Additional Supplementary Files [file 42003_2023_4510_MOESM2_ESM.pdf]

Description of Additional Supplementary Files

File name: Supplementary Movie
Description: Animation of paleotopography maps between 26,000 years ago to present.

File name: Supplementary Data
Description: Numeric data for Fig 2c, 3b, and 3c.
