# Supplementary figures and images for: Prehistoric human migration between Sundaland and South Asia was driven by sea-level rise
Source: Commun Biol. 2023 Feb 4;6:150. doi: 10.1038/s42003-023-04510-0 (PMC9899273; doi:10.1038/s42003-023-04510-0)

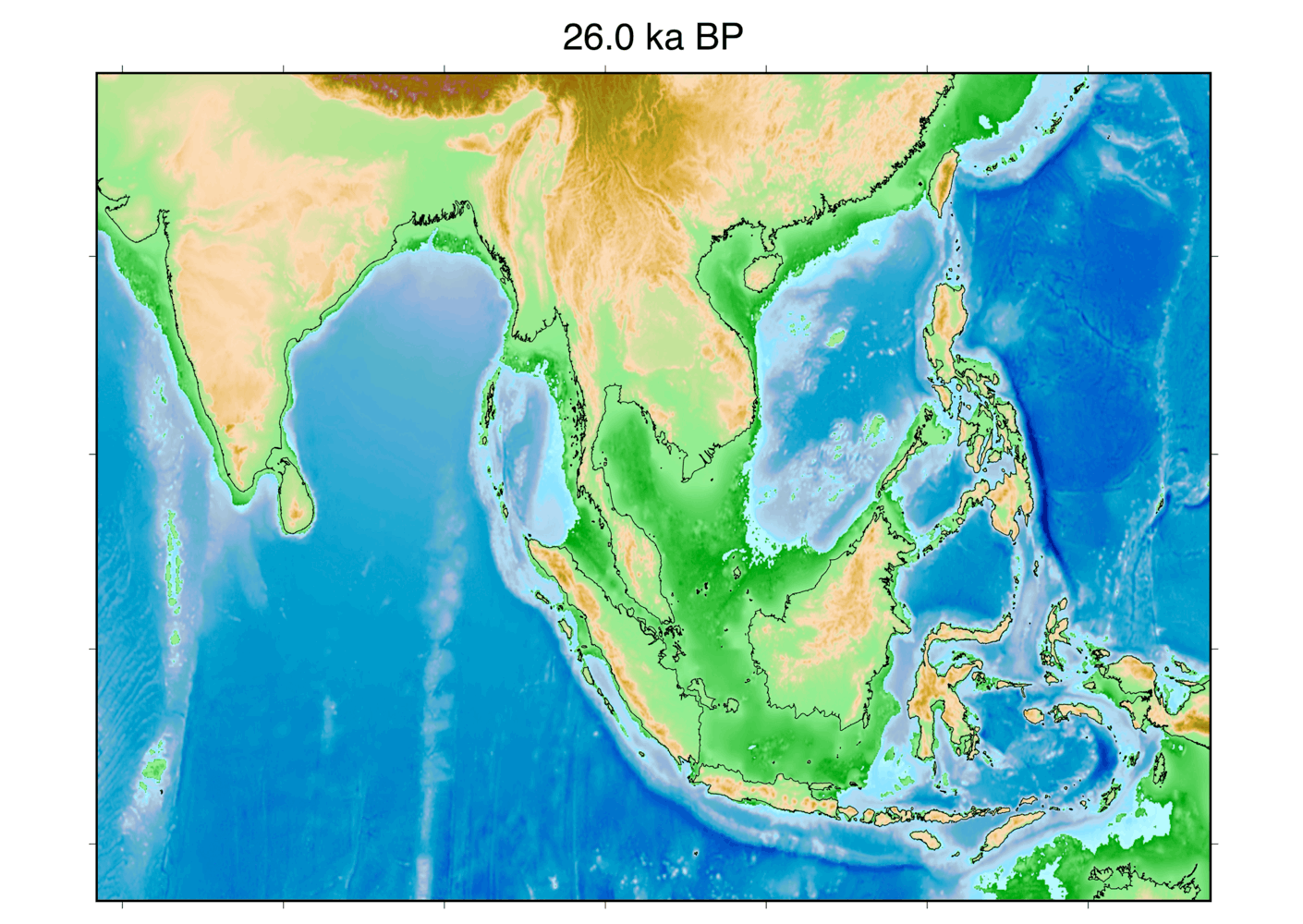

Supplement: Supplementary file 4 — Supplementary Movie [file 42003_2023_4510_MOESM4_ESM.gif]
